# Global Data From Great Ape Zoo Populations Confirm a High Prevalence of Overweight Individuals

**DOI:** 10.1002/ajp.70185

**Published:** 2026-07-12

**Authors:** João Pedro Meireles, Sarah Byrne, Paul W. Dierkes, Miriam Göbel, Max Hahn‐Klimroth, Arun Idoe, Dennis W. H. Müller, Zjef Pereboom, Jana Pluháčková, Sandra Reichler, Claudia Rudolf von Rohr, Simone Schehka, Jeroen M. G. Stevens, Jonas Verspeek, Marcus Clauss

**Affiliations:** ^1^ Clinic for Zoo Animals, Exotic Pets and Wildlife, Vetsuisse Faculty University of Zurich Zurich Switzerland; ^2^ Dublin Zoo Dublin Ireland; ^3^ Bioscience Education and Zoo Biology Goethe University Frankfurt Frankfurt Germany; ^4^ Zoo Zurich Zürich Switzerland; ^5^ Burgers, Zoo Arnhem The Netherlands; ^6^ Zoological Garden Halle Halle (Saale) Germany; ^7^ Antwerp Zoo Centre for Research and Conservation Antwerpen Belgium; ^8^ Zoo Ostrava Slezská Ostrava Czechia; ^9^ Tiergarten Heidelberg gGmbH (Zoo Heidelberg) Tiergartenstr Heidelberg Germany; ^10^ Westfälischer Zoologischer Garten Münster GmbH Münster Germany; ^11^ SALTO Agro‐ and Biotechnology Odisee University of Applied Sciences Sint Niklaas Belgium

**Keywords:** body condition, diet, *ex situ*, free‐ranging, growth, husbandry, obesity, primates, size, weight

## Abstract

Great apes in zoos have a history of receiving diets resembling those of humans in industrialized societies, with ensuing obesity and comorbidities. We analyzed adult zoo body mass (BM) data of 1290 chimpanzees, 141 bonobos, 231 Bornean orangutans, 186 Sumatran orangutans, and 719 Western gorillas in comparison to BM of free‐ranging conspecifics. Females and males considered overweight or obese were 62% and 45% in chimpanzees, 14% and 3% in bonobos, 88% and 81% in Bornean and 74% and 72% in Sumatran orangutans, and 67% and 12% in Western gorillas. The higher degree of overweight in females might also be linked to a lack of reproductive activity. Historical improvements, if at all, were evident in gorillas. We hypothesize that the main cause of obesity is the use of diet items that are low in fiber, high in easily digestible carbohydrates, and therefore highly palatable (such as cultivated fruit and pelleted or extruded compound feeds high in starch), instead of the recommended use of higher‐fiber items. As zoo animals do not choose diets for themselves but are provided with the items they can choose from, it is the responsibility of zoos to provide primates with diet items that can safely be used in a group setting without the incentive of overeating and the risk of monopolization by dominant individuals, that is, most plausibly, high‐fiber diets. For the optimal welfare of such long‐living taxa, the focus under human care should shift from sheer longevity to health span, for which the prevention of obesity is key.

## Summary

- We analyzed global body mass data from over 2500 zoo‐housed great apes across five species and compared them with free‐ranging conspecifics.
- Overweight and obesity are highly prevalent in zoo populations, particularly in chimpanzees, orangutans, and female gorillas, while bonobos show little deviation from wild body masses.
- Overweight is more extensive in zoo‐housed females, contributing to reduced body mass sexual dimorphism relative to wild populations.
- Historical trends show little improvement in most species, with notable reductions in overweight only in gorillas.
- We emphasize that diet composition—especially low fiber and high digestible carbohydrate intake—is a key, modifiable driver of obesity in zoo great apes.

## Graphical abstract

A global database reveals that a majority of great apes kept in zoos are overweight, likely as a result of the mismatch between their biology and their zoo diets. Corrective measures are necessary to reverse the situation and further improve the health and welfare of great apes in zoos.

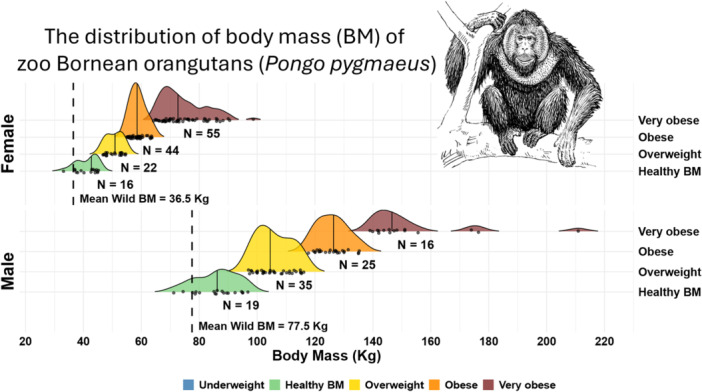

## 1 Introduction

Great apes are one of the most studied groups of wild mammals (Bezanson and McNamara 2019; Chen et al. 2023; Cronin et al. 2017; de Figueiredo Machado et al. 2023; Marshall et al. 2016; Shumaker 2018) and the most popular and studied animals in zoos (Binding et al. 2020; Edes 2023; Hopper 2017; Rose et al. 2019; Stoinski et al. 1998). This bias is due to their phylogenetic proximity to humans and charismatic appeal (Herzfeld 2017; Miralles et al. 2019). As such, they serve as valuable models for understanding human evolution, physiology, and cognition (Herzfeld 2017; Read et al. 2022; Tomasello and Herrmann 2010). Due to their cognitive proximity to humans, housing great apes in zoos has been controversial (Herzfeld 2017). Nevertheless, they are by far the group that has received the most directed efforts to improve animal care and life conditions over the last 50 years (Coe and Lee 1996; Hosey 2023; Hosey et al. 2020; Smith et al. 2014). Furthermore, all great ape species are at high risk of extinction, and zoo populations play a significant role in their conservation (Rietkerk and Pereboom 2018).

Numerous reports emphasize that great apes in zoos often suffer from the same so‐called “civilization diseases” as humans do: hypertension (Ely et al. 2013), diabetes (Kuhar et al. 2013), high cholesterol levels and cardiovascular disease (Dennis et al. 2019; McManamon and Lowenstine 2012; Schmidt et al. 2006; Strong et al. 2018; Strong et al. 2020; Van Mulders et al. 2024), and inflammatory disease (Nehete et al. 2014; Obanda et al. 2014), but the most widespread of all these comorbidities, often occurring alongside the above, is overweight and even obesity (Cocks 2007; Cousins 1972; Curry et al. 2023; Fooden and Izor 1983; Less 2012; Pontzer 2023; Videan et al. 2007). In humans, these pathologies are the manifestations of what is classified more broadly as the metabolic syndrome, resulting from the Western diet and lifestyle (Kopp 2019). Obesity, in particular, has been the most evident and widespread symptom of this syndrome (Engin 2024). In humans, this syndrome has been linked to poor health, lower quality of life, and shorter life expectancy (Busutil et al. 2017; Forhan and Gill 2013; Jia and Lubetkin 2005; Slagter et al. 2015). The susceptibility of great apes to obesity and its comorbidities does not only stem from their physiological similarities to humans (Pontzer 2023) but also from parallels in lifestyle observed in zoo‐housed great apes and industrialized human populations. It is worth noting that obesity in zoo animals is not exclusive to great apes and has been documented in other species (Goodchild and Schwitzer 2008; Krauss et al. 2026; Meireles et al. 2025; Schiffmann et al. 2020; Schwitzer and Kaumanns 2001).

In humans, obesity is strongly associated with diets high in saturated fats, sugars and starch, highly processed foods, and sedentary lifestyles (Kopp 2019). Likewise, great apes in zoos are more sedentary (Geijtenbeek et al. 2026), spend less time looking for food, and are often overfed with energy‐dense, low‐fiber foods that distinctively deviate from their natural diet (Less et al. 2014a; Smith et al. 2014; Smith et al. 2020; Van Mulders et al. 2024). The diets of great apes in the wild vary extensively by species, region, climate, and resource availability. Field studies often identify food items of various nutritional value using comparative terminology such as “high‐” or “low‐energy” to describe the value of diet items (Conklin‐Brittain et al. 1998; Lindshield et al. 2025; Vogel et al. 2025). Yet, when compared to diet items such as domesticated fruit, tubers, or starch‐based compound feeds, natural diets are composed mostly of more fibrous, lower‐energy plant matter. For instance, western gorillas consume large amounts of browse and fibrous plant matter, complemented by wild fruit and animal‐based items, when available (Lodwick and Salmi 2019; Remis et al. 2001; Remis 1997; Robbins et al. 2022), obtaining a considerable amount of their energy through fiber fermentation (Popovich et al. 1997). Similarly, during the long fruit scarcity periods, orangutans shift to leaves, vines, and bark, which are high in fiber and low in calories (Aguado et al. 2025; Tsutaya et al. 2022; Vogel et al. 2025). Chimpanzees display a very varied diet in the wild, as this is dependent on the habitat and population in question (Lindshield et al. 2025; McLennan and Ganzhorn 2017; Piel et al. 2017), but their fiber intake remains high, even during the fruit season (Conklin‐Brittain et al. 1998), since these resources are all of an indistinctively higher fiber content than domesticated fruit (Milton 1993). Chimpanzees are also known for their hunting capabilities and relatively higher consumption of animal protein than other apes, even sourcing it from mammal prey (Arcadi 2018; Fahy et al. 2013). However, existing feeding recommendations for primates in human care call for diets lower in fiber than those of their free‐ranging conspecifics in natural habitats (National Research Council 2003); people using these guidelines may be prone to just consider the numerical recommendations and not the biologically questionable reasons for not mimicking natural diets in this respect (Clauss et al. 2026). Thus, comparisons between natural environments and zoos provide a unique opportunity for primatologists to understand the outcomes of when diet and environment are in mismatch with the animal's biology, deepening our understanding of primate physiology, adaptation, and ecology.

Great ape husbandry has advanced considerably in recent decades (Coe and Lee 1996; Hosey 2023; Hosey et al. 2020; Talbot et al. 2023), and many breeding and longevity metrics have improved (Abello and Colell 2006; Havercamp et al. 2019; Porton and Niebruegge 2006; Rietkerk and Pereboom 2018; Strong et al. 2016; Wich et al. 2009). However, no long‐term evaluation of the body mass trends at the population level over time has been conducted. Body mass offers a useful indicator of the environment and care provided to zoo animals, and changes in this measure may reflect broader improvements in husbandry practices over time. This is especially important because, while solutions to tackle the problem are known (Cabana et al. 2018; Farmer et al. 2023; Less et al. 2014a), improvements may be hindered by lingering historical and cultural misconceptions about the diet of great apes, and often advanced knowledge and research regarding zoo primate nutrition is not emphasized enough at some zoos (Farmer et al. 2023; Fens and Clauss 2024). And thus, evaluations of historical trends can also inform primatologists about the readiness with which knowledge they accrue about the feeding ecology, natural diets, habitat seasonality, or primate physiology is incorporated into husbandry practices.

Here, we use the body mass data from the global zoo community via the Species360 Zoological Information Management System (ZIMS) database to examine the extent of the problem and to explore historical trends as well as patterns of seasonality and age. We are not the first to investigate the prevalence of obesity in zoo great ape populations: the works of Leigh (1994) and Pontzer (2023) have already evaluated how the body mass of zoo‐housed great apes compares with data from wild individuals. A comparison of their datasets and ours is shown in Table S1. Both followed a similar approach to ours regarding calculating average zoo body masses (asymptotic weight), but they only had access to publicly available datasets from zoo animals or veterinary reports, yielding a much smaller sample size.

Weight management and monitoring have become a cornerstone of zoo animal nutrition, health, and welfare (Clavadetscher et al. 2021; Meireles et al. 2025; Taylor et al. 2013). While such measures are highly valuable at the individual level for promoting health and welfare, evaluating and tracking body mass at the population level—as conducted in this study—provides a broader perspective on epidemiological trends, husbandry practices, and long‐term developments, and deepens our understanding of great ape biology.

## 2 Methods

### 2.1 Zoo Data

As part of the Species360 research data use agreement # 84212, we received data on body masses of great apes recorded in ZIMS and stored by Species360 in January 2023. Great ape zoo populations comprise the chimpanzee (*Pan troglodytes*), the bonobo (*Pan paniscus*), the Sumatran orangutan (*Pongo abelii*), the Bornean orangutan (*Pongo pygmaeus*), and the Western gorilla (*Gorilla gorilla*) (Fisken et al. 2018). Our data resolution was limited to the species level, which did not allow us to discriminate between subspecies or hybrids of (sub)species. Notably, the zoo chimpanzee population is composed of pure individuals of all four subspecies and hybrids (Fisken et al. 2018). Hybrids of orangutan (*abelii* × *pygmaeus*) are also present in the zoo population (Fisken et al. 2018; Göbel and Venzke 2024), as they were once considered the same species, but these hybrids are not represented in our data, as these individuals are not identified to species level. However, we could not control for instances of mislabeled individuals, since this is the responsibility of each zoo when recording their animal data.

The entire dataset was anonymized, indicating only the body mass entered by a zoo, the date of record, a randomly assigned individual animal ID, sex, and the corresponding age of the animal, but not the identity of the reporting zoo. Hence, we could not discriminate among zoos within a spectrum of different husbandry standards. However, given the membership fee to belong to Species360 and licensing the ZIMS software, and the effort required to routinely weigh the individuals and enter the data in the database, it is likely that the vast majority of data originates from high‐standard facilities that prioritize their animal husbandry and records. The zoo body mass data derived from the ZIMS database did not contain information on feeding protocols, health records, reproductive histories, or activity data. Consequently, potential drivers or consequences of the observed patterns, such as caloric intake, diet composition, activity regimes, medication, and comorbidities, could not be evaluated in this analysis. The dataset represents, in most cases, sequential body mass records of the same individual across its lifespan (e.g., Figure S2); in other words, single biased weight values due to disease or pregnancy events are unlikely. The dataset comprises both historical and current data, including both deceased and living individuals. These raw data were provided with an indication of which data points were considered outliers by several automated correction procedures; additionally, manual correction of remaining outliers was applied, as explained in Garand et al. (2024) and Meireles et al. (2025).

### 2.2 Wild Body Mass Data

Data on the adult body mass of wild adult great apes were taken from the scientific literature and from online museum collections whose records include the body mass of the specimens that provided material for the museums (Tables S1–S3). Leigh (1994) and Smith and Jungers (1997) extensively discuss the issues and limitations behind the use of literature extraction of wild body masses, regarding sample size, individuals, and seasonal variations, inaccuracies in age estimation, and weighing methodology. Furthermore, being aware of these limitations, we followed these rules when accepting wild body mass records:

- Excluding individuals in rehabilitation, in sanctuaries, or supplementarily‐fed.
- Excluding individuals imported from the wild if the measurement was taken at arrival at the zoo.
- Accepting road kills if the measurement was taken while the carcass was still fresh and in good condition.

We defined an average wild body mass per sex for each species by calculating the weighted average of the multiple literature sources. When referring to the species’ average wild body mass (including both sexes), the average between the male and female weighted averages was used. For chimpanzees, given the distinct difference in body mass and available literature between subspecies, the weighted average was done first per subspecies and later the average of the three considered subspecies was used for analysis. We considered this the most conservative approach, given the indeterminate taxonomy of our data for chimpanzees. The different outcomes for the analysis if each subspecies was selected as the benchmark can be seen in Figure S5.

### 2.3 Analysis

For each species, for females and males separately, a Gompertz growth model was fitted to the zoo data. This model yields an asymptotic weight that can be interpreted as the growth plateau (Zullinger et al. 1984). Note that Gompertz models may not necessarily be the best models to fit growth data (reviewed in Veylit et al. (2021)); here, we did not employ them to yield the most accurate data fit, but only to define the age at which animals typically reach adult size (=adult age; as defined and discussed by Smith and Jungers (1997)). The adequacy of the models was checked by inspecting the resulting model as plotted against the raw data (Figure S1). We defined the age from which on data would be included in the calculation of an adult average as 95% of this asymptotic mass. The resulting parameter estimates and ages used as the cut‐off to define adulthood size are given in Table 1.

**Table 1:** Results of Gompertz model fit (according to $y=A\;{e^{-e}}^{-k\left(t-t_{0}\right)}$) to the age‐specific body mass data of females and males of great ape species kept in zoos, and the resulting threshold age for defining adulthood.

Species	Sex	Asymptote mass (*A*; kg)	Time to maximum growth (*t*_*0*_, years)	Relative growth rate (*k*; d^−1^)	Threshold age (years)
*Pan troglodytes*	Female	55.9 (±0.1)	3.1 (±0.0)	0.3 (±0.0)	15.9
	Male	63.3 (±0.1)	5.2 (±0.2)	0.3 (±0.0)	13.7
*Pan paniscus*	Female	38.7 (±0.1)	3.6 (±0.1)	0.3 (±0.0)	14.0
	Male	46.4 (±0.1)	3.7 (±0.1)	0.3 (±0.0)	15.2
*Pongo abelii*	Female	52.4 (±0.1)	3.4 (±0.1)	0.2 (±0.0)	17.9
	Male	108.3 (±0.4)	13.0 (±0.8)	0.3 (±0.0)	19.5
*Pongo pygmaeus*	Female	63.7 (±0.1)	2.9 (±0.0)	0.2 (±0.0)	21.0
	Male	114.1 (±0.3)	6.6 (±0.2)	0.2 (±0.0)	20.9
*Gorilla gorilla*	Female	96.5 (±0.1)	3.4 (±0.0)	0.3 (±0.0)	14.5
	Male	183.3 (±0.2)	8.5 (±0.1)	0.3 (±0.0)	16.5

Abbreviations: *A* = asymptotic adult body mass, *k* = relative growth rate, *t*_*0*_ = time until maximum growth.

Based on the detected adult age threshold, the body mass was averaged per individual and then across the means of all individuals. After confirming the normal distribution of the sex‐specific body mass data of males and females using the Shapiro–Wilk normality test, we tested for sexual dimorphism in the zoo population by an independent *t*‐test in case *p* (Shapiro–Wilk) > 0.05, and otherwise by a Mann–Whitney *U* test using Python's scipy package. Next, we calculated the average body mass of each individual per year. Following a previous approach (Krauss et al. 2026; Mellor et al. 2020; Taylor et al. 2012), we classified individuals into body mass categories based on how much the adult average body mass deviates from the weighted average body mass in the wild (determined by the values that we found in the literature). An individual is considered underweight if it has 0.75 or less of the wild average body mass. Between 0.75 and 1.25 times the wild body mass, an individual is considered to have a healthy body mass. Between 1.25 and 1.5, it is considered overweight; between 1.5 and 1.75, obese; and above 1.75, very obese. We depicted this classification of body masses chronologically, from the first year with at least 10 individuals with data, calculating the proportion of the difference scores in the population to determine developments over time.

Additionally, two patterns were assessed visually by analyzing the data for each individual animal separately: (i) regular, annual (i.e., seasonal) fluctuations in body mass, and (ii) a decrease in body mass towards later adult life. As a first step, only those individuals were selected for which at least three measurements in both the first part of the expected lifetime as well as the second part of the expected lifetime were present. To assess seasonal fluctuations, pairs of measurements between one winter and the subsequent summer (or vice versa) were used. Finally, the data of these pre‐selected individuals were plotted and all inspected individually, assessing subjectively if the detected pattern was visually evident (then it was counted) or not (then it was not). Examples of such individual plots are given in Figure S2. Using this information, we determined the proportion of these individuals that showed a seasonally fluctuating body mass and a decline in body mass with progressing age late in adult life.

## 3 Results

The statistics and age thresholds resulting from the Gompertz models can be seen in Table 1. Sources for the body mass of wild adult great apes derive mostly from museum specimens (individuals shot and later taxidermized), and in some cases, different publications refer to the same museum specimens, resulting in an overlap of the data. Body masses of wild living animals are scarcer (Rayadin and Spehar 2015; Uehara and Nishida 1987). Some species, particularly gorillas, bonobos, and Sumatran orangutans, have low sample sizes for wild body mass. No literature source mentioned or discriminated for body masses of pregnant or infant‐carrying females. Sex‐specific zoo data for all species are given in Table 2, and a comparison to wild data and a classification into body mass categories are given in Table 3.

**Table 2:** Body mass records (in kg) and patterns for adult, zoo‐kept great ape species in the present study. Note that this selection of individuals is based on data availability and may not be representative of the current global zoo populations.

Species	*n*	Mean ± SD (min, max)	Male:Female ratio	% seasonal fluctuations (of *n*)^a^	% old age decline (of *n*)^a^
Sex					
*Pan troglodytes*					
Female	741	54.6 ± 10.8 (26.0, 93.2)	1.11 (a)	4.7% (43)	42.3% (26)
Male	552	60.8 ± 9.9 (35.5, 105.8)		0.0% (11)	28.6% (7)
*Pan paniscus*					
Female	81	37.0 ± 5.2 (25.4, 51.9)	1.21 (A)	0.0% (8)	20.0% (5)
Male	60	44.9 ± 6.1 (30.0, 64.0)		0.0% (4)	25.0% (4)
*Pongo abelii*					
Female	118	54.3 ± 11.7 (27.2, 85.0)	1.92 (A)	0.0% (11)	18.1% (11)
Male	68	104.2 ± 23.3 (36.1, 161.5)		0.0% (10)	10.0% (10)
*Pongo pygmaeus*					
Female	137	61.8 ± 13.0 (33.1, 98.7)	1.85 (A)	0.0% (22)	28.6% (14)
Male	94	114.2 ± 21.8 (71.2, 176.4)		0.0% (14)	37.5% (8)
*Gorilla gorilla*					
Female	403	96.3 ± 16.7 (52.7, 170‐0)	1.85 (a)	2.9% (68)	35.6% (45)
Male	316	178.7 ± 26.5 (103.0, 274.0)		3.4% (59)	43.2% (37)

*Note:* A/a indicate significant sexual dimorphism, as assessed by a parametric *t*‐test (A) or a nonparametric *U*‐test (a) in the case of not normally distributed data.

^a^ Number of individuals with sufficient data quality for the given assessment.

**Table 3:** Summary of average wild and zoo body masses and the percentage (%) of individuals in each body mass category.

Species	Sex	N (zoo)	Mean Wild BM^a^	Mean Zoo BM	Zoo/Wild	Under‐weight	Healthy BM	Over‐weight	Obese	Very obese
*P. troglodytes*	Female	741	40.3	54.6 ± 10.8	1.36	0.3	**37.6**	36.4	16.7	9.0
	Male	552	49.0	60.8 ± 9.9	1.24	0.3	**54.7**	34.1	9.0	2.0
*P. paniscus*	Female	81	33.6	37.0 ± 5.2	1.10		**86.4**	12.3	1.2	
	Male	60	46.5	44.9 ± 6.1	0.97	3.3	**93.3**	3.3		
*P. abelii*	Female	118	37.3	54.3 ± 11.7	1.46	0.8	25.8	**28.0**	27.1	18.6
	Male	68	74.8	104.2 ± 23.3	1.39	2.9	25.0	**36.8**	19.1	16.2
*P. pygmaeus*	Female	137	36.5	61.8 ± 13.0	1.69		11.7	16.1	32.1	**40.1**
	Male	94	77.5	114.2 ± 21.8	1.47		20.2	**37.2**	26.6	17.0
*G. gorilla*	Female	403	70.7	96.3 ± 16.7	1.36	0.2	32.5	**43.9**	16.6	6.5
	Male	316	165.9	178.7 ± 26.5	1.08	1.9	**86.1**	10.1	1.9	

*Note:* Bold values represent the largest group in each sex.

^a^ Weighted average, calculated from literature sources (Tables S1–S3).

### 3.1 Pan troglodytes

Chimpanzees in the wild vary in mass depending on the subspecies in question, but globally, their body mass varies between 23 and 50 kg for females (*n* = 87) and 32 and 60 kg for males (*n* = 82) (Table S1). Males are 22% heavier than females (M:F ratio 1.22).

**Figure 1:**
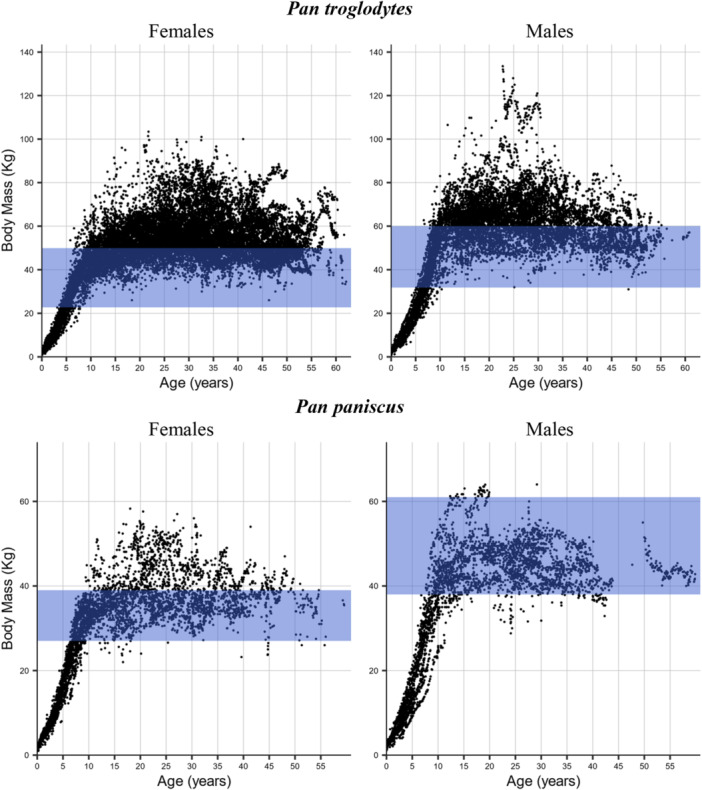
Body mass data for zoo‐kept *Pan* spp. (black dots) as compared to the literature data range of adult, free‐ranging specimens (blue) (for sources, see Table 2). Note that this selection of individuals is based on data availability and not necessarily representative for the current global zoo populations.

**Figure 2:**
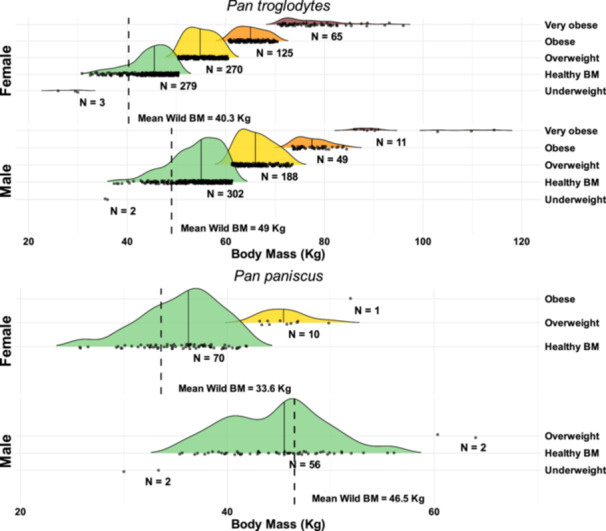
Distribution of densities of body mass scores across *Pan troglodytes* and *Pan paniscus* individuals in zoos.

For the global zoo population of chimpanzees, the manually corrected dataset contained 741 female and 552 male individuals (Table 2). From the Gompertz models, female and male chimpanzees under human care are estimated to obtain adult body size when 13.7 and 15.9 years old, respectively (Table 1). The average body mass of zoo‐kept animals is 55 (±11) kg for females and 61 (±10) kg for males (Table 2). Males are significantly heavier than females (11% on average, M:F ratio 1.11), but this disparity is smaller than observed in the wild (Table S1). A large proportion of our data for both sexes is well above the range of body masses of wild specimens (Figure 1). In fact, 62% of females are categorized as being overweight or heavier. While most males are classified as having healthy body mass (55%), 45% of them are overweight or heavier (Table 3, Figure 2). The average body mass of a zoo chimpanzee is 36% heavier than the average in the wild for females, and 24% for males (Table 3).

Depending on which chimpanzee subspecies is chosen as the reference for wild body mass, the proportion of overweight animals ranges from a maximum of 93% (females) and 85% (males) if *P.t. schweinfurthii* is the basis for comparison to a minimum of 40% (females) and 9% (males) if *P.t. troglodytes* is the basis for comparison (Figure S5). Thus, even if the latter subspecies is chosen as the reference, the zoo population still contains a relevant number of female individuals considered overweight.

### 3.2 Pan paniscus

Female bonobos in the wild range between 27 and 39 kg (*n* = 13), and males range between 38 and 61 kg (*n* = 13). In bonobos, males are about 38% heavier than females (M:F ratio = 1.38, Table S1).

For bonobos, the corrected zoo dataset contained 81 female and 60 male individuals (Table 2) with body mass data above 14.0 and 15.2 years of age for each sex, respectively (Table 1). The average body mass of zoo bonobos was 37 (±5) kg for females and 45 (±6) kg for males (Table 2). Males are 21% heavier than females in zoos (M:F ratio 1.21); a smaller difference than that of data from the wild (Table S1). While some females in zoos stand above the range found in the literature for wild specimens, males in zoos seem to display a body mass that is typical of males in the wild (Figure 1). Both sexes have a large proportion of individuals within a healthy body mass: 86.4% and 93.3% for females and males, respectively (Table 3, Figure 2). Zoo females are 10% heavier than their wild counterparts, whereas zoo males are 3% lighter (Table 3).

### 3.3 Pongo spp.

Most body masses found in the literature for male orangutans do not discriminate between flanged and unflanged males, although it is known that these two morphotypes differ greatly in body mass (Marty et al. 2015; Scott et al. 2024). Only Rayadin and Spehar (2015) provide separate measurements between these males for Bornean orangutans (Table S2). Since all measurements from other sources fall above the range of unflanged males from Rayadin and Spehar (2015), we considered them as flanged males. For the Sumatran orangutan, we did not find sources that allow a similar differentiation, but it may be safe to extrapolate that the variation between males follows a similar pattern (Dunkel et al. 2013). Adult female Sumatran orangutans in the wild (*n* = 9) range between 34 and 45 kg, while males (*n* = 5) stand between 54 and 86 kg (Table S2). For the Bornean orangutan, females range between 25 and 48 kg in the wild (*n* = 31), while (flanged) males range between 58 and 97 kg (*n* = 26). The unflanged males from Rayadin and Spehar (2015) range between 28 and 58 kg, falling mostly within the body mass range of the females, as expected. In the Bornean orangutan, (flanged) males are 112% heavier than females in the wild, while for Sumatran orangutans, males are 101% heavier than females (M:F ratios of 2.12 and 2.01, Table 3).

**Figure 3:**
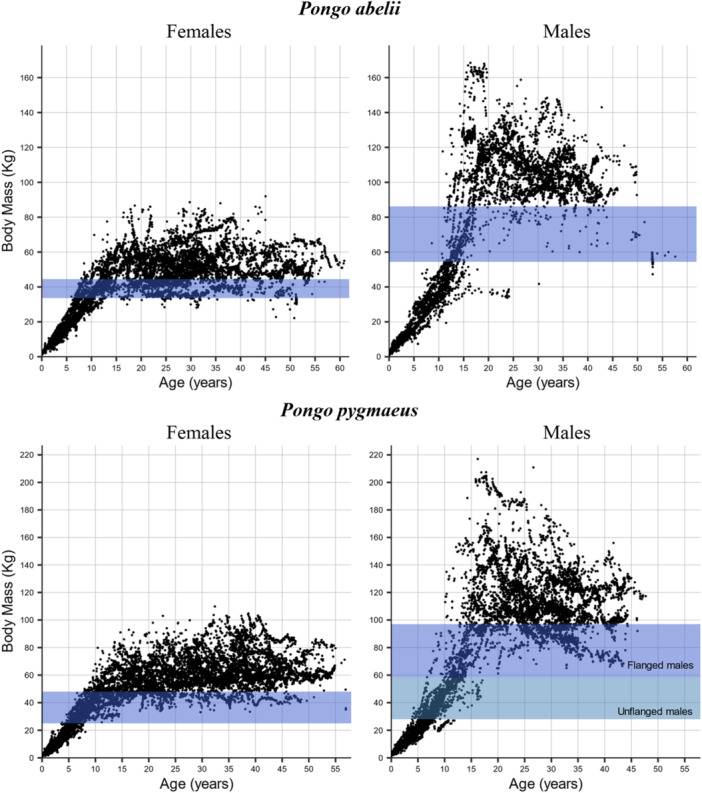
Body mass data for zoo‐kept *Pongo* spp. (black dots) as compared to the literature data range of adult, free‐ranging specimens (blue) (for sources, see Table 2). Note that this selection of individuals is based on data availability and is not necessarily representative of the current global zoo populations.

**Figure 4:**
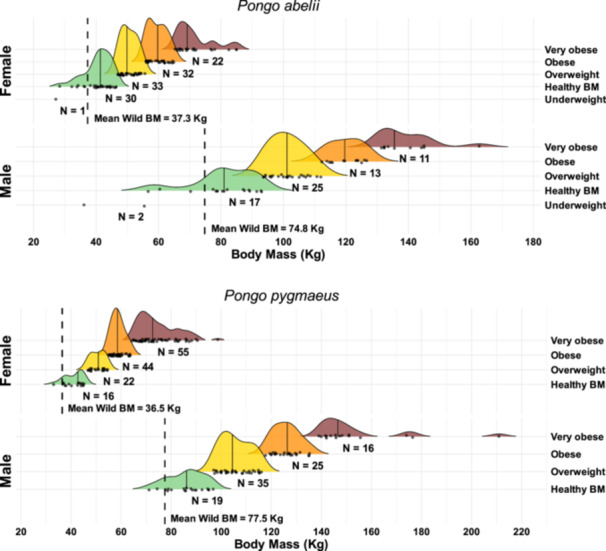
Distribution of densities of body mass scores across *Pongo abelii* and *Pongo pygmaeus* individuals in zoos.

The zoo dataset of Sumatran orangutans contained 118 female and 68 male individuals (Table 2) with data above 17.9 and 19.5 years of age (Table 1). In zoos, females on average weigh 54 (±12) kg and males 104 (±23) kg, with this difference being significant (Table 2). While the sexual dimorphism of body mass is similar to that observed in the wild (92%—Table S2), both sexes are well above the typical body mass found in the literature for wild individuals (Figure 3). Twenty‐five per cent of individuals are classified as having a healthy body mass in both sexes, with the remainder classified as overweight or obese (Table 3, Figure 4). The average female in zoos is 46% heavier than in the wild, and the average male is 39% heavier (Table 3).

The assessment of 137 female and 94 male zoo individuals of Bornean orangutan (Table 2) with data above 17.9 and 19.5 years of age (Table 1) shows that, similarly to Sumatrans, most zoo individuals weigh well above the range of body masses described in the literature for free‐ranging specimens (Figure 3). Eleven per cent of females are classified as being of a healthy body mass, and almost half of the females are classified as very obese (40.1%) (Table 3, Figure 4). In zoos, females on average weigh 62 (±13) kg and males 114 (±22) kg. Males are significantly heavier than females (85% on average, Table 3), but this magnitude of dimorphism is smaller in zoos than observed in the wild (Table S2). Males of typical body mass seen for unflanged males in the wild are nearly absent in zoos, with a single *P. abelii* specimen in this range (Figure 3). The average female Bornean orangutan in zoos is 69% heavier than in the wild, while for males this discrepancy is 47% (Table 3).

### 3.4 Gorilla gorilla

**Figure 5:**
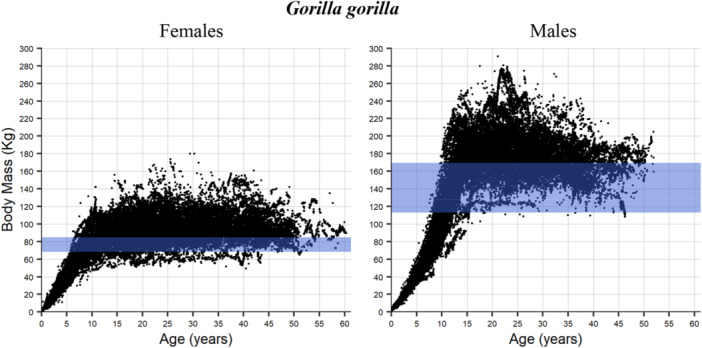
Body mass data for zoo‐kept *Gorilla gorilla* (black dots) as compared to the literature data range of adult, free‐ranging specimens (blue) (for sources, see Table 2). Note that this selection of individuals is based on data availability and not necessarily representative for the current global zoo populations.

**Figure 6:**
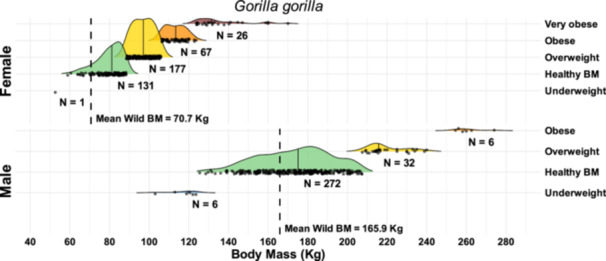
Distribution of densities of body mass scores across G*orilla gorilla* individuals in zoos.

Female western lowland gorillas (*n* ≥ 5) in the wild range between 68 and 85 kg, while males (*n* ≥ 17) range between 112 and 170 kg. An adult male gorilla in the wild is, on average, 135% heavier than a female (M:F ratio 2.35, Table S3). The assessment of 403 female and 316 male zoo gorillas (Table 2) with data above 14.5 and 16.5 years of age (Table 1) reveals that many zoo individuals weigh above the range described in the literature for free‐ranging specimens (Figure 5). The vast majority of male zoo gorillas are within a healthy body mass (86.1%), but many females are overweight (43.9%), obese (16.6%) or very obese (6.5%) (Table 3, Figure 6). In zoos, females, on average, have a body mass of 96 (±17) kg and males 179 (±27) kg. Males are significantly heavier than females (85% on average, Table 2), but this magnitude of dimorphism is smaller in zoos than observed in the wild (Table S3). The average female gorilla in zoos is 36% heavier than in the wild, while the average zoo male is only 8% heavier than in the wild (Table 3).

### 3.5 Historical Trends

**Figure 7:**
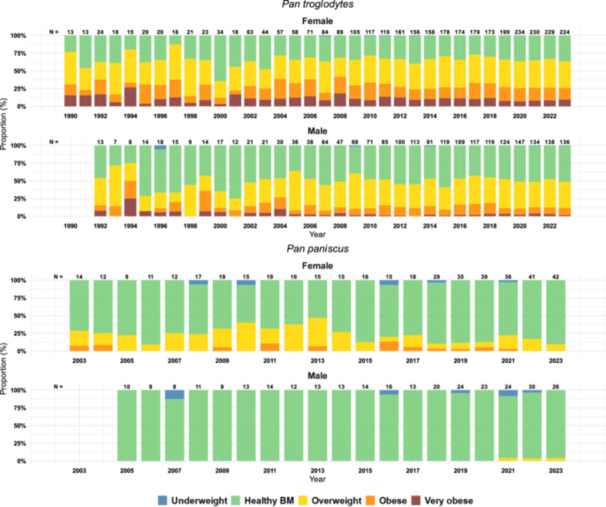
Historical trends (by year) of body mass category proportions in adult zoo‐kept *Pan troglodytes* and *Pan paniscus*. Data were used from the first year in which a minimum of 10 individuals were present in the dataset. Note that this selection of individuals is based on data availability (individuals weighed) and is not necessarily representative of the current global zoo populations.

**Figure 8:**
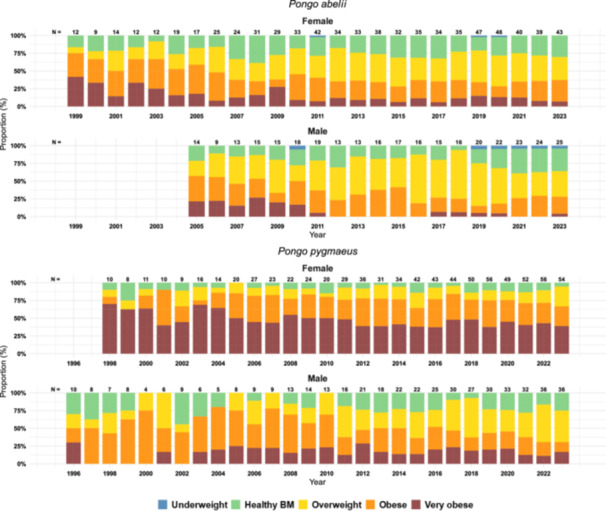
Historical trends (by year) of body mass category proportions in adult zoo‐kept *Pongo abelii* and *Pongo pygmaeus*. Data were used from the first year in which a minimum of 10 individuals were present in the dataset. Note that this selection of individuals is based on data availability (individuals weighed) and is not necessarily representative of the current global zoo populations.

**Figure 9:**
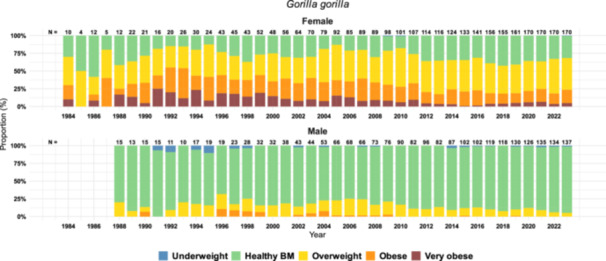
Historical trends (by year) of body mass category proportions in adult zoo‐kept *Gorilla gorilla*. Data were used from the first year in which a minimum of 10 individuals were present in the dataset. Note that this selection of individuals is based on data availability (individuals weighed) and is not necessarily representative of the current global zoo populations.

The historical developments of body mass in great apes have robust data since 1985 for the Lowland gorilla, 1990 for the chimpanzee, and between the late 90 s and early 2000s for the remaining species (bonobos and orangutans) (Figures 7, 8, 9). Both chimpanzees and bonobos have a stable pattern of body mass categories over time (Figure 7). Neither orangutan species displays notable trends over time regarding the proportion of obese and very obese individuals, with the exception of male Sumatran orangutans, where, in recent times, there appears to be a reduction in the number of individuals who are overweight or obese (Figure 8). For gorillas, the proportion of individuals of higher body masses (overweight, obese and very obese) for both sexes seems to have a downward trend over time (Figure 9).

### 3.6 Seasonal and Age Patterns

Overall, for all species of great ape, nearly no individual displayed a seasonal pattern of body mass fluctuations (Table 3). No species displayed a remarkable decline in body mass with age (> 50% of the individuals), with mostly only a small number of individuals doing so; 43.2% of male gorillas and 42.3% of female chimpanzees, with appropriate data, show a decline in body mass with age (Table 3).

## 4 Discussion

**Figure 10:**
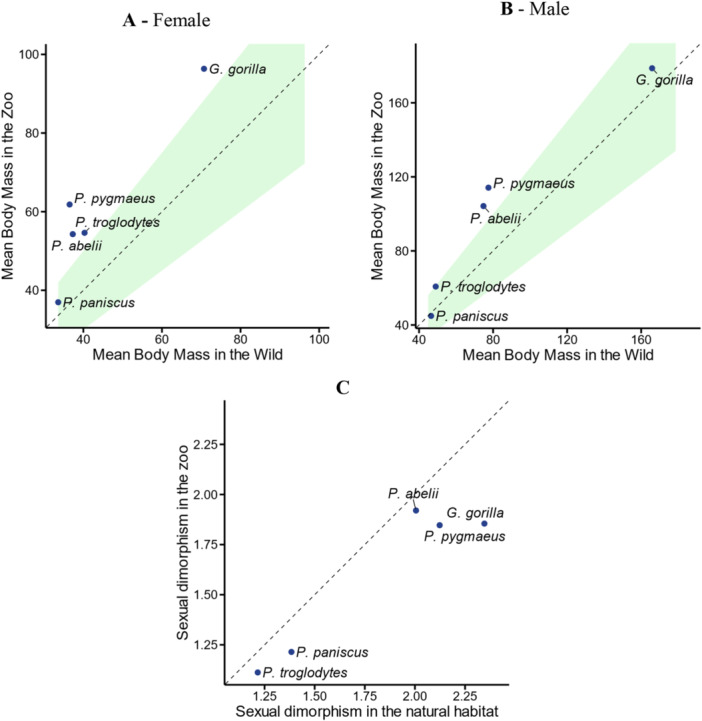
Relationships between the zoo body mass and characteristics of the Great Apes. (A and B)– species comparison between the body mass in the zoo and in wild populations. The green area represents a range of 25% around the wild body mass. (C)– variation of body mass sexual dimorphism (as the ratio of male:female mean body mass) between zoo and wild.

**Figure 11:**
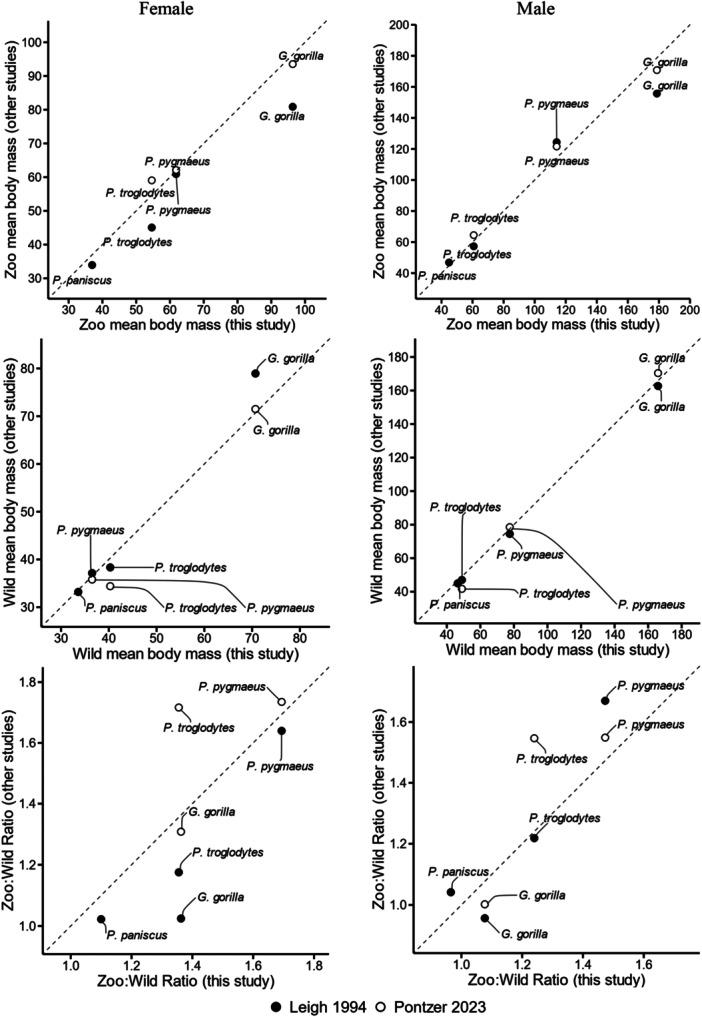
Comparison of the data evaluated in the present study with two previous surveys of zoo primate body mass (Leigh 1994; Pontzer 2023).

Great apes kept under human care have already been described for a long time as generally overweight or obese (Cocks 2007; Cousins 1972; Fooden and Izor 1983; Videan et al. 2007). Our study further corroborates this description, with most species, except the bonobo and male gorillas, being systematically heavier than the typical body mass described for wild specimens (Figure 10AB) and having a large number of individuals that can be considered obese or very obese (Table 3). Notably, females in zoos are more often overweight than males, as also revealed by the diminished sexual dimorphism seen in zoo populations (Figure 10C). Our results do not differ greatly from previous studies that compared wild and zoo populations (Leigh 1994; Pontzer 2023) (Table S4, Figure 11). The most noteworthy exception is the female gorilla, which Leigh (1994) described as being overweight to a lesser extent than in our study. This stems from differences with respect to both the zoo and the wild data. Our zoo gorilla population is heavier than the data in Leigh (1994), and the wild body mass used as a reference by him was higher than ours (whereas that used by Pontzer (2023) was similar to our wild data) (Figure 11). Furthermore, Leigh (1994) described that males display a larger difference compared to the wild, suggesting that males are at higher risk of obesity, but in our case, females seem to be the sex that deviates more from the wild body masses and in which obesity is more prevalent (Figure 11).

### 4.1 Limitations

Our dataset was limited to body mass records available from the scientific literature for free‐ranging specimens and from Species360 for zoo animals. Evidently, more data, especially on free‐ranging specimens, would be welcome. However, it should be noted that it was not those species for which the largest samples of free‐ranging specimens have been published (chimpanzees, Bornean orangutans) that were classified as least overweight in this study, indicating that a larger comparative sample would not automatically change the outcome. Among the zoo specimens, with only 81 females and 60 males, bonobos were the species with the smallest sample size analyzed, most likely due to the generally lower numbers of this species in zoos. While we believe that this, as well as the larger samples, are likely to be representative of the zoo population, this could only be proven if all zoo animals were weighed repeatedly.

The zoo data was anonymized by design, and there were no corresponding institutional metadata (feeding plans, social groups, activity records, reproductive state, medication, or disease history). Therefore, while the observed differences in body mass between zoos and natural habitats are robust as descriptive population‐level patterns, our data cannot, on their own, establish causal pathways driving those patterns, but require biologically plausible, hypothetical explanations.

Furthermore, the dataset does not allow an evaluation of the physical status of the individuals beyond body mass. Not accounting for body condition can be considered limiting and simplistic (Turner et al. 2016; Van Mulders et al. 2024), because it is not possible to distinguish between the effect of excess adiposity and optimized growth potential with body mass alone. This limitation is present in most assessments of zoo animal obesity (Garand et al. 2024) and acknowledged to various degrees in the primate literature (Mellor et al. 2020; Pontzer 2023; Terranova and Coffman 1997).

In humans, the effects of nutrition and disease status on body height, and hence also body mass, have been well described: under better nourishment and under fewer disease challenges, humans realize their full growth potential to a higher degree (Floud et al. 2011). Therefore, without accompanying measures of body size and without the context of the environmental status, body mass data alone cannot formally differentiate between an increase in skeletal body size (i.e., “unrestrained growth”) or an accretion of body fat. There has been evidence that captive animals develop morphometrical differences from their wild counterparts (O'Regan and Kitchener 2005; Siciliano‐Martina et al. 2021), including body size, in which their nutritional environment is one factor at play (Geiger 2021; Hanegraef and Spoor 2024; O'Regan and Kitchener 2005; Turner et al. 2016). It has been suggested that chimpanzees and orangutans grow larger in captive settings than in the wild (Fooden and Izor 1983; Hanegraef and Spoor 2024; Kimura and Hamada 1996), and data on a higher amount of muscle mass in zoo than in free‐ranging orangutan females (Harwell et al. 2026) point in the same direction. Yet, other studies with chimpanzees, other primates, and humans (Altmann et al. 1993; Cole et al. 2020; Turner et al. 2016; Walker et al. 2006) describe a strong link between body mass and adiposity. For example, Turner et al. (2016) described that wild and captive vervet monkeys (*Chlorocebus aethiops*) did not differ in morphometric measures, but captive individuals were heavier and in better body condition than wild individuals. Furthermore, a review on human physical growth has described that while height potential, over several generations, inevitably reaches a plateau, the body mass of most human populations continues to rise due to the obesity epidemic (Cole 2003). We could not control for this distinction, but we expect differences to be mainly related to body fat, not size. While we share this methodological uncertainty with previous work, individual experiences with zoo animals (e.g., Supp. Figures S3 and S4) do suggest that differences in body mass will, to a relevant extent, reflect degrees of obesity.

A further limitation concerns the potential influence of pregnancy on the female average body mass. Our dataset, covering the body mass records of individuals across their lifespans, did not include information on pregnancy events, and therefore, we could not exclude data points related to pregnancy periods from the analyzes. Pregnancy can contribute to elevated body mass in placental mammals; however, the increase in body mass resulting from a pregnancy in a female great ape is relatively small compared to its absolute body mass and occurs briefly, given that only in the later stages of gestation, a significant increase in weight does occur (~ 4–2 last months of gestation)(Grether and Yerkes 1940). The effect of this brief increase is diluted when averaging data records of an entire individual's lifespan across decades. For instance, on average, orangutan newborns are 1.73 kg (Fooden and Izor 1983), newborn gorillas are 2.0 kg (Bellisari et al. 2001; Ruff et al. 2022), and newborn chimpanzees are 1.8 kg (Fessler et al. 2005; Grether and Yerkes 1940), representing only ~2%–4% of the mother's mass in non‐human great apes (DeSilva 2011). Placental tissues are likewise low in weight, at less than 1 kg (Soma 1983).

Thus, data from late pregnancy alone would theoretically not push a female of average wild body mass into the “obese” category. Empirical observations are consistent with this assumption: Grether and Yerkes (1940) tracked the body weight of 31 female chimpanzees across pregnancy, and on average, they gained 5.3 kg in the last 2 months of gestation. This represents a 13% increase compared to the average body mass of an adult female from their sample (40.8 kg), when pregnancy body masses were excluded. Furthermore, a female gorilla at Yerkes Primate Research Center only increased 12% in body mass across its pregnancy (Riddle et al. 1973), whereas a female at Krefeld Zoo became leaner during gestation (Meder 1986). Furthermore, we believe that breeding females are not more intensively monitored than non‐breeding females or that the body mass of females is more intensively monitored during gestation than in non‐gestational life periods, as it is seen by the equal frequency and effort that exists in body mass monitoring of males in the dataset, suggesting no bias towards reproductive events. As a result, while pregnancy may introduce additional inflation into the body mass of females, we expect its impact on the overall population‐level averages to be limited and unable to be the main factor behind the magnitude of 35%–70% disparity between zoo and wild body masses of females.

Theoretically, many biases could apply to the data used. Hypothetically, one could claim that zoos selectively weigh those individuals whose body mass is of concern, that is, overweight individuals, and cease monitoring them once a normal body mass has been reached. But even if that were the case, the large sample size of the present study would still translate into a high prevalence of overweight that did not change noticeably in historical time.

### 4.2 Potential Causes for Obesity

The underlying causes of the prevalence of overweight in zoo great apes have been highlighted for decades (Cousins 1972). Great apes in the wild have diets that are high in fiber and low in sugars and starches (Galdikas 1988; Harrison et al. 2010; Hohmann et al. 2010; Remis et al. 2001; Robbins et al. 2022; Rogers et al. 2004) while that of zoos has traditionally often been high in cereals or their byproducts, cultivated fruit, nuts, oily seeds and even dairy products (e.g., yoghurt) and meat, delivering fat and easily digestible carbohydrates (Crandall 1964; Dierenfeld 1997; Less et al. 2014a; Less et al. 2014b; Ruempler 1992; Schmidt et al. 2005; Smith et al. 2014; Van Mulders et al. 2024). For instance, the bulk of food items consumed by gorillas in the wild are fibrous plants, and they consume much smaller amounts of wild fruit and animal protein (Lodwick and Salmi 2019; Robbins et al. 2022), but Crandall (1964) and Ruempler (1992) report that gorillas in zoos traditionally would regularly receive cultivated fruit, meat, milk, fruit juice, dog pellets, nuts, and cooked rice, all items that are higher in digestible energy than their natural diet.

The effects of the consumption of human food (agricultural crops) are even evident in free‐ranging specimens. The McLennan and Asiimwe (2016)'s instance of a road‐killed female chimpanzee living in an area where wild chimpanzees complement their wild diet with human crops is a good example. This female was heavier than what is typical for a female of the species and had more adipose tissue than normal. A further investigation of the nutritional composition of the diet of chimpanzees in the area showed that these crops had a significantly higher amount of easily digestible carbohydrates and were poorer in fiber than the wild diet of chimpanzees (McLennan and Ganzhorn 2017). A similar scenario has also been reported in baboons (Altmann et al. 1993).

Most great apes are evolutionarily adapted to environments that are prone to fluctuations in food resources (Knott 2005). For instance, tropical rainforests tend to have marked dry and wet seasons that strongly correlate with the brief fruit production of most plant species (Corlett and Primack 2011). Taking advantage of this intermittent availability of an energy‐dense food resource requires adaptations such as fat storage and fat mobilization, which are essential for reproduction success (Knott 2005; Pond 1997; Pontzer et al. 2014). These adaptations may explain the extreme weights achieved by most orangutans in zoos, for instance. Orangutans have a particularly slow metabolism—one of the lowest among primates (Pontzer et al. 2010) ‐ which, in combination with a diet that is readily consumed in excess of energetic needs, easily leads to adipose tissue accretion. In addition, their energy expenditure might also be reduced, as it has been described that zoo orangutans move less than their wild counterparts (Geijtenbeek et al. 2026). In the wild, this slow metabolism enables them to quickly store fat during the short period of mast seeding of fruit trees, which occurs only every 2–7 years. These unusually infrequent fruiting events are unique to Southeast Asian rainforests (Corlett and Primack 2011). During fruit‐scarce periods, individuals must resort to consuming low‐energy fibrous plant matter and become leaner (Knott 1998; O'Connell et al. 2021). Western gorillas are also seasonal frugivores, taking advantage of the short fruiting season, while eating mostly fibrous plant materials the rest of the year (Remis 1997) and might, to a lesser extent, have a similar ability to store fat during times of abundance (Zihlman and McFarland 2000). It is worth noting that different populations may show considerable variations in their diets, reflecting variations in habitat, environmental seasonality, available food items, and even habits (McGrew 1983; Robbins et al. 2022; Serckx et al. 2015). Seasonal fluctuations in food quality and food quantity, and the corresponding seasonal body condition/weight fluctuations seen in the wild are, according to our results, mostly absent in zoos (Table 2). If great apes have evolved to go through long cycles of eating only low‐energy foods, zoo diets with a constant supply of sugars and starches are poorly aligned to their physiology.

Furthermore, reproductive management may also be a contributing factor to the generalized overweight in the populations, especially in females. Oral contraception is widely used in zoos for great apes (Agnew et al. 2016; Nederlof et al. 2024; Sarfaty et al. 2012). While there is no evidence either in humans or primates that oral hormonal contraception increases the risk of weight gain (Edelman et al. 2011; Gallo et al. 2014; Lopez et al. 2016), other contraception methods, like some implants, can cause weight gain in zoo animals (Cowl et al. 2025). Reproduction is an energy‐costly core activity of an individual's life, and removing it removes an important long‐term stimulus for activity (Schiffmann et al. 2025). Lactation is the most energetically costly phase of reproduction in primates (Dufour and Sauther 2002; Hassler et al. 2024; Pontzer et al. 2014; Thompson et al. 2012)—mothers may increase food intake, change time budgets, or mobilize fat stores to meet the high costs of milk production and infant care (Altmann and Samuels 1992; Guedes et al. 2008; Lappan 2009; Thompson et al. 2012). Future research analyzing the potential impact that contraception has on the body mass or body condition of zoo great apes will be welcome.

Great apes are known to be sexually dimorphic in many aspects, including in body size, and that pattern is equally seen in the zoo populations, but contrary to what is seen in some other species, where males grow distinctly bigger in zoos (Dinerstein 1991; Meireles et al. 2025), the sexual dimorphism between males and females in great apes seems to be reduced (Figure 10C). This is noticeable by the lower M:F ratios observed in zoo populations than those seen in the wild, and how females exceed the typical body mass of the species to a larger degree than males (Figure 10A). In humans, it has been described that the female body is more prone to adipose storage than the male body, since adipose reserves are important to sustain female reproduction (Koceva et al. 2024; Power and Schulkin 2008), and it has been similarly suggested for great apes (Knott 2005; Zihlman and Bolter 2015). And in baboons (*Papio cynocephalus*), it has been observed that female body condition is more strongly affected by high caloric diets than that of males (Altmann et al. 1993).

### 4.3 Species‐Specific Considerations

The two orangutan species differ in the extent to which they deviate from the mean wild body mass. Bornean orangutans are heavier and more frequently obese than Sumatran orangutans when compared to wild specimens (Figures 3, 4). A speculative explanation for this might come from the fact that Bornean orangutans cope much less than Sumatran orangutans with social group living in zoos. This has been measured by comparison of the cortisol levels of zoo animals of the two species (Weingrill et al. 2011). Not only is chronic cortisol exposure hypothesized to contribute to obesity (Chao et al. 2017; Jackson et al. 2017; Ma et al. 2022), but we suspect that Bornean orangutan groups are often provided with more food as a measure to pacify social tensions.

The near absence of overweight or obese individuals in our dataset of bonobo zoo individuals is in line with what was previously described by Zihlman and Bolter (2015). They found a negligible amount of body fat in their sample of 13 zoo bonobos and state that male bonobos do not store fat easily, even under optimal nutrition, whereas females exhibit body fat fluctuations linked to reproduction while remaining relatively lean. This reduced propensity to fat storage may also be linked to the lower nutritional stress that bonobos face in their natural habitat, when compared to chimpanzees (Oelze et al. 2016b; White and Wood 2007). Bonobos occur in habitat areas of evergreen forest that are tendentially more stable and less seasonal than chimpanzee habitats (Oelze et al. 2016a; Oelze et al. 2016b). Additionally, we hypothesize that, given the lower rank of males in female‐dominated bonobo troops (Vervaecke et al. 2000), males might be less successful in securing first access to preferred (energy‐dense) food items in a group feeding context compared to other great ape species (Parish 1994; White and Wood 2007). Nevertheless, there are individual cases of obese bonobos, particularly a male used for research called Kanzi, which was fed with sugary drinks and candy for decades and reached a body weight of 91 kg (Ape Initiative 2025; Wong 2025). We suspect that Kanzi was part of our initial dataset, but given his extreme body mass, he was removed as an outlier (see supporting materials Figures S3 and S4).

All great apes are susceptible to the undesired behavior of regurgitation and reingestion (R/R), and in all species, it has been shown that a reduction of cultivated fruit in zoo diets is a measure to reduce its occurrence (Cabana et al. 2018; Hill 2018). However, a higher prevalence or a higher awareness in gorillas as compared to other apes, together with a higher (real or perceived) relevance of wild fruit in the natural diets of chimpanzees and orangutans, may have made zoos more compliant with the call for a reduction of cultivated fruit and an increase in roughage‐based foraging material in gorillas. For example, whereas the number of studies on R/R in gorillas is large (Akers and Schildkraut 1985; Barrett et al. 2025b; Fuller et al. 2018; Gould and Bres 1986a; Gould and Bres 1986b; Hill 2009; Less et al. 2014a; Lukas 1999; Remis and Dierenfeld 2004; Ruempler 1992; Smith et al. 2020; Tennant et al. 2022), corresponding studies in chimpanzees (Baker 1997; Baker and Easley 1996; Bloomsmith et al. 1988; Mulder et al. 2016; Struck et al. 2007; Wallace et al. 2019), bonobos (Miller and Tobey 2012; Stevens and Wind 2011) or orangutans (Cassella et al. 2012; Choo et al. 2011; Nash et al. 2021) are more limited. Thus, while speculative, this extensive focus on gorilla feeding regimens to address R/R may explain the relatively lower number of overweight or obese individuals in our results (Table 3) and may have driven the visible reduction in the proportion of overweight or obese individuals in the last few decades in this species (Figure 9).

### 4.4 Body Mass Senescence

Age‐related body mass senescence has been described in many mammal species (Douhard et al. 2017; Hämäläinen et al. 2014; Hamrick et al. 2006; Kroeger et al. 2018; Nussey et al. 2011; Tafani et al. 2013; Weladji et al. 2010), in baboons (Alberts et al. 2014), and humans (Forbes and Reina 1970). The reduction in body mass has been linked to loss of muscle (lean) mass (sarcopenia) (Demontis et al. 2013) and to a reduction in bone density due to a reduction in activity levels (Hamrick et al. 2006). While great apes and humans share many age‐related health issues, humans are more prone to sarcopenia and osteoporosis (Lowenstine et al. 2016). If great apes tend to lose physical condition with age, it is not yet widely established. Harwell et al. (2026)'s model did not establish age as a predictor of lean body mass in orangutans. On the other hand, Pusey et al. (2005) have described that wild male chimpanzees lose weight with age, while females do not, and this is corroborated to some extent by the results of Thompson et al. (2020) on lean mass loss. In our results, some chimpanzees of both sexes displayed a degree of body mass loss with ageing, which was more frequent in females than in males. The differing results in our assessment from those of previous studies may be a result of sampling bias or due to the overweight in many chimpanzee individuals, masking the decline with age. It is relevant to note that loss of body mass with age increases the risk of obesity on its own, since it is driven by a decrease in muscle(lean):fat ratios, activity levels, and metabolic rate, as described for humans and dogs (Harper 1998). However, Thompson et al. (2020) did not find a reduction of activity levels with age in wild chimpanzees. Given the long longevity attained by great apes in zoos, it becomes even more relevant to monitor and control their body condition/mass and diet needs. Allowing individuals to remain obese throughout their lifespan and develop related comorbidities goes against the responsibility of guaranteeing optimal welfare for animals in zoos.

### 4.5 Consequences of Obesity

Obesity has the potential to impact general welfare as obese individuals may suffer from other comorbidities, and the excessive body mass restricts functional mobility, hindering the capacity to perform a wider range of natural behaviors for the species. It is known that in humans, obese individuals have a lower quality of life, beyond compromised physical and psychological health, driven by difficulties in performing daily physical activities and reduced endurance (Forhan and Gill 2013). There is already a large number of publications describing health issues in great apes related to obesity (see Introduction), including a reduction in longevity (Cocks 2007). Thus, obesity can severely impact the health and welfare of the individuals and make the care and veterinary management of great apes more costly and complex.

Obesity may also create negative implications for reproduction. In humans, obesity has been linked to reproductive problems and a reduction in fertility of both sexes (Catalano and Ehrenberg 2006; Cogswell et al. 2001; Esposito and Giugliano 2005). For instance, obese women are at a higher risk of infant mortality and parturition complications (Chen et al. 2009; Stubert et al. 2018), and a similar link between obesity and offspring survival has been described in cattle and pigs (Li et al. 2019; Trzebiatowski et al. 2025). Although no research has been done to date linking obesity with reproductive problems in great apes, the risk seems plausible. Given that sustaining the reproductive health of great apes in zoos is fundamental for their *ex situ* conservation, lowering the incidence of obesity should be taken as a priority. Furthermore, increased body weight might trigger puberty in young great apes earlier than expected, just as it happens with humans (Biro et al. 2012), rhesus macaque (Terasawa et al. 2012), and zoo elephants (Glaeser et al. 2012). There are examples of other mammal breeding programs, such as for elephants, rhinoceroses, or pandas, where obesity or overweight has been pointed out as negatively affecting reproduction (Chusyd et al. 2018; Edwards et al. 2015; Tang et al. 2023; Zhou et al. 2025).

### 4.6 Solutions to Prevent Obesity in Great Apes

Our historical overview of each population reveals that there has been limited change in the management of body mass in great apes. Only the gorilla population seems to display a small improvement in reducing the proportion of individuals in higher body mass categories. This is despite the findings of Cocks (2007) that overweight female orangutans have a shorter lifespan (a pattern most likely true for the other species too), and despite ongoing updates of husbandry guidelines on diets (AZA Ape TAG 2017; AZA Gorilla SSP 2017; Bemment 2018; Bemment et al. 2024; Carlsen et al. 2022; Meinelt et al. 2025), demonstrating that there is still considerable work to be done for compliance with diet recommendations.

Nevertheless, significant improvements have been achieved in the last 50 years in zoo primate nutrition, with a reduction in animal products and overall energy density (Cabana et al. 2018; Moittié et al. 2022; Smith et al. 2014; Viallard et al. 2023). Further significant improvements are still needed to achieve the widespread use of diets that could be considered safe with respect to their propensity to trigger obesity. Interestingly, behavioral abnormalities like R/R (see above) may have been more distinct drivers of great ape diet changes than obesity‐related problems, although this must remain speculative.

Arguably, the most important factor for controlling obesity in zoo primates would be to raise dietary fiber levels into the vicinity of natural diets (Clauss et al. 2026). Lower fiber levels, and corresponding higher levels of easily digestible carbohydrates, are a hallmark of the diets of many zoo primates (Cabana et al. 2018; Plowman and Cabana 2019). This discrepancy has been well‐known for a long time (e.g., Oftedal and Allen 1996; Van Mulders et al. 2024), and it has even been proactively defended by the National Research Council (2003), which recommends lower fiber levels for captive primates compared to those ingested in the wild, even though dietary changes towards higher‐fiber diets have generally resulted in either positive or at least no negative consequences (reviewed in Clauss et al. 2026). In group‐fed animals such as great apes, feeding high‐energy items in a restricted way is not considered safe, because dominant individuals may exert aggression to monopolize more than their share, leaving subordinate animals malnourished. Rather, offering sufficient amounts of less palatable (aka “healthy”) diet items that are not ingested in excess is recommended; such diets do not compromise body weight maintenance in healthy individuals or growth of juveniles, but reduce the incentive to overeat and hence the prevalence of obesity (Plowman 2013). And due to the lower incentive to overeat, that is, the lesser presence of preferred items, aggression linked to feeding events is typically also reduced as there is “less to fight about” (Britt et al. 2015; Viallard et al. 2023). Additionally, avoiding a mix of diet items at any single feeding event, even when all items are classified as high‐fiber, has recently been considered an important measure to reduce monopolization of preferred items, reducing any incentive for overconsumption and aggression. Thus, it is recommended that at any feeding event, only one kind of vegetable (e.g., only celery or only chard) should be provided.

Evidently, increasing the fiber levels of zoo diets would imply, for many zoos, abandoning the inclusion of cultivated fruit in primate diets. The use of certain compound feeds would also need to be discontinued, as these feeds—although sometimes marketed as “high‐fiber”—contain significantly less fiber than what is technically achievable and far less than what primates consume in their natural diets. For example, there is a tradition in commercial zoo animal diets that those formulated for folivorous primates (typically called “leaf‐eater,” with crude fiber levels below 15% but nevertheless labeled “high‐fiber”; MC, pers. obs.) often have distinctively lower fiber levels than those formulated for folivorous hoofstock (typically called “browser,” with crude fiber levels ≥ 20%; MC pers. obs.), even though the natural diet of these groups does not suggest such a systematic difference. Hence, a change in compound feeds away from low‐fiber towards truly high‐fiber products is warranted; note that this is not a general rejection of compound feeds, but a call for zoos to use and buy, and hence for producers to offer, compound feeds for primates made using the current technical capacities to produce truly high‐fiber products. Additionally, forage provision—specifically, browse—would have to be increased. Our findings might indicate a relative inertia in making the corresponding changes (Fens and Clauss 2024), even though they are outlined in Best Practice Guidelines (Bemment 2018; Bemment et al. 2024; Carlsen et al. 2022; Stevens 2020). This may well be due to the fact that great apes and humans alike prefer cultivated fruit over vegetables when given a choice (Offringa et al. 2019; Remis 2002; Verspeek and Stevens 2020), and that a switch from a low‐fiber to a higher‐fiber compound feed will predictably be met with resistance by the animals, and hence resistance from zoos to implement them in diet plans.

Another essential component of obesity prevention in zoo‐housed great apes is the reduction of sedentary behavior (Leitzmann 2017; Rimbach and Pontzer 2024). A necessary complement to dietary reform is the systematic promotion of species‐appropriate physical activity. By redesigning feeding strategies and husbandry so that animals must search for, travel to, extract, and manipulate food (e.g., through foraging devices, puzzle feeders, suspended or spatially distributed feeders, and unpredictable feeding schedules), institutions can effectively reduce passive consumption and increase locomotion and activity levels (Dishman et al. 2009; Sha et al. 2015) thus, increasing daily energy expenditure. The provision of the total daily diet in ways that access is challenging (but not overtaxing) for the animals has been identified as one of the hallmarks of modern zoo animal feeding programs (Fens and Clauss 2024).

The implementation of dietary regimes for great apes in zoos that follow the recommendations listed above is a plausible next step that should not be delayed by calls for further research. Whenever diet changes are made as recommended, and the logistical situation allows, careful monitoring of various measurements are welcome, including detailed records of diet protocols before and after the change, offered versus consumed diet quantities, body mass, body condition score or skinfold thickness developments, fecal consistency, feeding behavior and activity budgets, endocrine profiles including corticoid metabolites, reproductive and metabolic hormones such as leptin or urinary C‐peptide, documentation of the fecal (and oral) microbiome, and direct measures of body fat by ultrasound or dual energy X‐ray absorptiometry. However, the fact that any or all of these measurements are not possible should not prevent dietary changes that are plausible and have been safely made in other facilities (Barrett et al. 2025a; Cabana et al. 2018; Less et al. 2014a; Less et al. 2014b; Smith et al. 2020).

### 4.7 Conclusions

Great apes in zoos typically achieve longer lifespans than their wild counterparts (Che‐Castaldo et al. 2021; Havercamp et al. 2019; Wich et al. 2009), and such long lifespans bring new challenges regarding welfare and veterinary care. As has been argued for human populations (Gostin and Garsia 2014; Olshansky 2018; Wickramasinghe et al. 2020), the focus should shift from the increase of absolute longevity to guaranteeing that the lives of these animals are spent in good health. Aging increases the risk of health and welfare decline (Lowenstine et al. 2016), which is amplified if coupled with obesity and inadequate nutrition. Preventing obesity (and associated comorbidities) (Lowenstine et al. 2016) is an essential component of promoting health span and welfare throughout an ape's life.

Given the currently alarming and growing obesity epidemic seen around the world in human populations (Bixby et al. 2019; Phelps et al. 2024; Rodriguez–Martinez et al. 2020), where the root causes are known, and the health and societal costs are ever‐growing, it is worrying to see a broad presence of the phenomenon in populations of zoo great apes, too. This parallel extends further into the solutions to reverse the situation (implementation of a proper nutritional environment in both cases), and more concerningly, insufficient corrective actions. Funding for projects like “the great ape heart project,” trying to address the main cause of mortality in great apes in zoos (Murphy and Danforth 2019; Murphy et al. 2018), is available to tackle the end‐of‐the‐line consequences, while action still lags when it comes to changing the diets of great apes in zoos and preventing obesity in the first place.

## Author Contributions

**João Pedro Meireles:** conceptualization, formal analysis, methodology, visualization, writing – original draft, writing – review and editing, data curation. **Sarah Byrne:** writing – review and editing. **Paul W Dierkes:** writing – review and editing, supervision. **Miriam Göbel:** writing – review and editing. **Max Hahn‐Klimroth:** methodology, software, data curation, formal analysis. **Arun Idoe:** writing – review and editing. **Dennis W. H. Müller:** writing – review and editing, conceptualization. **Zjef Pereboom:** writing – review and editing. **Jana Pluháčková:** writing – review and editing. **Sandra Reichler:** writing – review and editing. **Claudia Rudolf von Rohr:** writing – review and editing, funding acquisition. **Simone Schehka:** writing – review and editing. **Jeroen M. G. Stevens:** writing – review and editing. **Jonas Verspeek:** writing – review and editing. **Marcus Clauss:** writing – review and editing, writing – original draft, conceptualization, methodology, supervision.

## Ethics Statement

The authors have nothing to report.

## Conflicts of Interest

The authors declare no conflicts of interest. This study is intended as a constructive assessment to support evidence‐based husbandry improvements; the authors are practitioners and scientists within the zoo community committed to animal health and welfare.

## Supporting information


Supporting File 1

Supporting File 2

## Data Availability

The data that support the findings of this study are available in the supporting information of this article.
