# Supplementary material for: Global Data From Great Ape Zoo Populations Confirm a High Prevalence of Overweight Individuals
Source: Am J Primatol. 2026 Jul 12;88(7):e70185. doi: 10.1002/ajp.70185 (PMC13358328; doi:10.1002/ajp.70185)
Supplement: Supplementary file 1 — Supporting File 1 [file AJP-88-e70185-s002.docx]

***Supplementary material***

**Global data from great ape zoo populations confirms that overweight individuals are the norm**

**João Pedro Meireles, Max Hahn-Klimroth, Dennis W. H. Müller, Zjef Pereboom, Claudia Rudolf von Rohr, Miriam Göbel, Jana Pluháčková, Tjerk ter Meulen, Paul W. Dierkes, Marcus Clauss**

**Tables S1-S4**

**Figures S1-S4**

**Table S1** Body mass records (in kg) for wild adult *Pan* sp.

| **Source** |  | ***Female*** |  |  |  | ***Male*** |  | **M:F ratio** |
| --- | --- | --- | --- | --- | --- | --- | --- | --- |
|  | **n** | **Mean ±SD (range)** | |  | **n** | **Mean ±SD (range)** | |  |
| *Pan troglodytes* |  |  |  |  |  |  |  |  |
|  |  |  |  |  |  |  |  |  |
|  | *P. t. troglodytes* | | |  |  |  |  |  |
| **Smith and Jungers (1997)** | 2 | 41.0 | |  | 1 | 59.3 | | 1.45 |
| **Jungers and Susman (1984)** | 3 | 47.4  (36.6-43.0) | |  | 3 | 60 | | 1.27 |
|  | *P. t. verus* | | |  |  |  |  |  |
|  |  |  |  |  |  |  |  |  |
| **Isler et al. (2008)**  Supplementary material | 1 | 49.9 | |  | 1 | 46.3 | | - |
|  |  |  | |  |  |  | |  |
| **Smith and Jungers (1997)** | 3 | 41.6 | |  | 1 | 46.3 | | 1.11 |
|  | *P. t. schweinfurthii* | | |  |  |  |  |  |
| **Smithsonian NMNH^1^** and **Isler et al. (2008)** Supplementary material | 1 | 31.3 | |  | - | - | | - |
|  |  |  |  |  |  |  |  |  |
| **Morbeck and Zihlman (1989)** | 6 | 29.8  (26.4-32.3) | |  | 9 | 39.5  (33.6-47.3) | | 1.33 |
|  |  |  | |  |  |  | |  |
| **Jungers and Susman (1984)** | 19 | 33.2 | |  | 15 | 43.0 | | 1.30 |
|  |  |  |  |  |  |  |  |  |
| **Uehara and Nishida (1987)** | 8 | 35.2 ±3.9  (30.0-41.8) | |  | 6 | 42.0 ±5.4  (34.3-49.6) | | 1.19 |
|  |  |  | |  |  |  | |  |
| **Wrangham and Smuts (1980)** | 6 | 29.8 ±2.2  (22.7-35.5) | |  | 9 | 39.5 ±4.5  (31.8-49.5) | | 1.33 |
|  |  |  | |  |  |  | |  |
| **Rahm (1967)** | 9 | 34.3 ±5.6  (27.6-46.0) | |  | 3 | 42.8 ±2.8  (41.0-46.0) | | 1.25 |
|  |  |  | |  |  |  | |  |
| **Pusey et al. (2005)** | 26 | 31.3 | |  | 31 | 39.0 | | 1.25 |
|  |  |  | |  |  |  | |  |
| **McLennan and Asiimwe (2016)^2^** | 1 | 40.0 | |  | - | - | | - |
|  |  |  | |  |  |  | |  |
| **Smith and Jungers (1997)** | 3 | 35.9 | |  | 3 | 53.7 | | 1.50 |
|  |  |  |  |  |  |  |  |  |
| *Pan paniscus* |  |  |  |  |  |  |  |  |
|  |  |  |  |  |  |  |  |  |
| **Jungers and Susman (1984) via Leigh & Shea (2003)** | 6 | 33.2 | |  | 7 | 45 | | 1.36 |
|  |  |  | |  |  |  | |  |
| **Morbeck and Zihlman (1989)** | 6 | 33.4  (27.0-38.5) | |  | 4 | 45  (38.0-61.0) | | 1.35 |
|  |  |  | |  |  |  | |  |
| **Coolidge and Shea (1982)** | 1 | 37.0 | |  | 2 | 54.5 ±6.5  (48.0-61.0) | | 1.47 |
|  |  |  |  |  |  |  |  |  |

^1^https://collections.nmnh.si.edu/search/mammals/

^2^this female was not considered when calculating the average wild body mass for the species, as she feeds on human crops.

**Table S2** Body mass records (in kg) for wild adult *Pongo* spp.

| **Source** |  | ***Female*** |  |  |  | ***Male*** |  | **M:F ratio** |
| --- | --- | --- | --- | --- | --- | --- | --- | --- |
|  | **n** | **Mean ±SD (range)** | |  | **n** | **Mean ±SD (range)** | |  |
| *Pongo abelii* |  |  |  |  |  |  |  |  |
|  |  |  |  |  |  |  |  |  |
| **Smithsonian NMNH^1^** and **Isler et al. (2008)** Supplementary material | 6 | 37.3 ±3.4  (33.6-44.5) | |  | 4 | 72.0 ±13.2  (54.4-86.2) | | 1.93 |
|  |  |  | |  |  |  | |  |
| **Markham and Groves (1990)** | 2 | 38.3 ±3.0  (36.6-43.0) | |  | 1 | 86.2 | | 2.25 |
|  |  |  | |  |  |  | |  |
| **Wich et al. (2004)** | 1 | 35.7 | |  | - | - | | - |
|  |  |  |  |  |  |  |  |  |
| *Pongo pygmaeus* |  |  |  |  |  |  |  |  |
|  |  |  |  |  |  |  |  |  |
| **Isler et al. (2008)**  Supplementary material | 11 | 36.9 ±4.3  (31.8-45.4) | |  | 6 | 80.6 ±9.7  (67.0-90.7) | | 2.18 |
|  |  |  |  |  |  |  | |  |
| **Rayadin and Spehar (2015)**  live-caught individuals |  |  |  |  |  | *Flanged Males* | |  |
|  | 7 | 35.3 ±6.8  (25.0-48.0) | |  | 12 | 74.0 ±9.0  (58.0-97.0) | | 2.10 |
|  |  |  |  |  |  | *Unflanged Males* | |  |
|  |  |  |  |  | 5 | 42.2±10.8  (28.0-58.0) | |  |
|  |  |  | |  |  |  | |  |
| **Lyon (1911)** | 4 | 38.8 ±4.6  (32.7-45.4) | |  | 4 | 85.6 ±5.2  (79.4-90.7) | | 2.21 |
|  |  |  | |  |  |  | |  |
| **Smithsonian NMNH^1^** | 9 | 36.0 ±4.2  (32.0-45.0) | |  | 4 | 75.0 ±5.9  (79.0-91.0) | | 2.10 |
|  |  |  | |  |  |  | |  |
|  |  |  |  |  |  |  |  |  |

^1^https://collections.nmnh.si.edu/search/mammals/

**Table S3** Body mass records (in kg) for wild adult *Gorilla gorilla*

| **Source** |  | ***Female*** |  |  |  | ***Male*** |  | **M:F ratio** |
| --- | --- | --- | --- | --- | --- | --- | --- | --- |
|  | **n** | **Mean ±SD (range)** | |  | **n** | **Mean ±SD (range)** | |  |
| *G. g. gorilla* |  |  |  |  |  |  |  |  |
|  |  |  |  |  |  |  |  |  |
| **Willoughby, D. P. (1950)** | nr | 85 | |  | nr | 156 | | 1.83 |
| **Burgess et al. (2018)** | 1 | 68.2 | |  | 2 | 140.4 ±27.7  (112.7-168.0) | | 2.05 |
| **Jungers and Susman (1984)** | 3 | 71.5 | |  | 14 | 169.5 | | 2.37 |
|  |  |  |  |  |  |  |  |  |

**Table S4** Comparison between this study and previous studies analysing body masses of great apes in zoos and in the wild.

| **Source** | **This study** | | |  | **Leigh (1994)** | |  | **Pontzer (2023)** | | |
| --- | --- | --- | --- | --- | --- | --- | --- | --- | --- | --- |
|  | N Zoo | Zoo mean | Relative zoo:wild |  | Zoo mean | Relative zoo:wild |  | N Zoo | Zoo mean | Relative zoo:wild |
| *Pan troglodytes* | 1290 | 57.69 | 1.29 |  | 42.68 | 1.20 |  | 302 | 61.77 | 1.62 |
| *Pan paniscus* | 141 | 40.93 | 1.02 |  | 39.1 | 1.03 |  |  |  |  |
| *Pongo abelii* | 186 | 79.25 | 1.41 |  |  |  |  |  |  |  |
| *Pongo pygmaeus* | 231 | 87.99 | 1.54 |  | 55.81 | 1.66 |  | 36 | 91.85 | 1.61 |
| *Gorilla gorilla* | 719 | 137.51 | 1.16 |  | 120.82 | 0.98 |  | 58 | 132.15 | 1.09 |

| \| 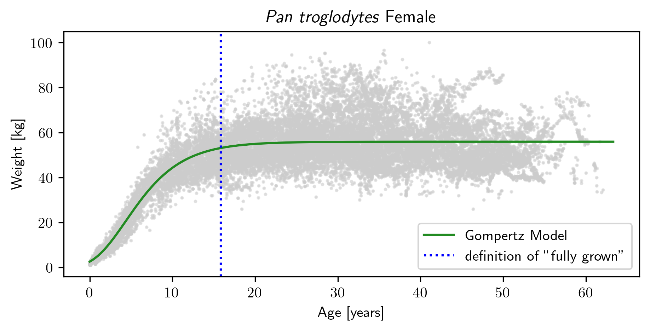 \| 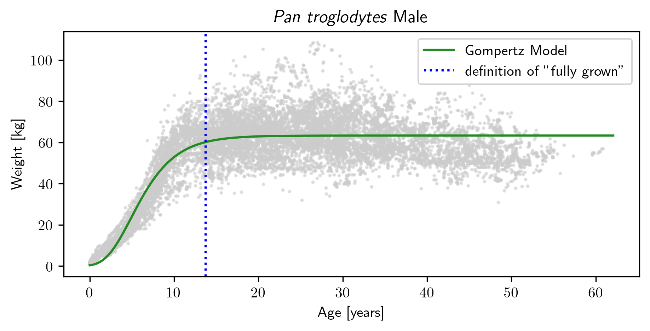 \| \| --- \| --- \| \| 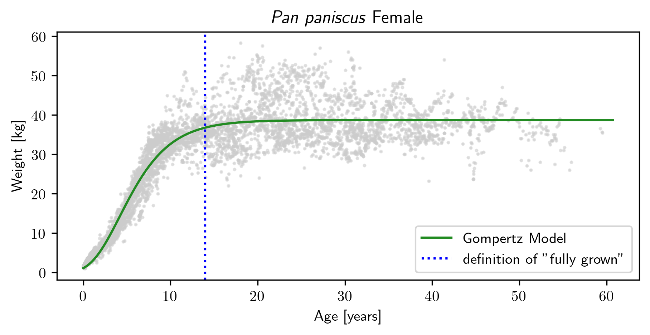 \| 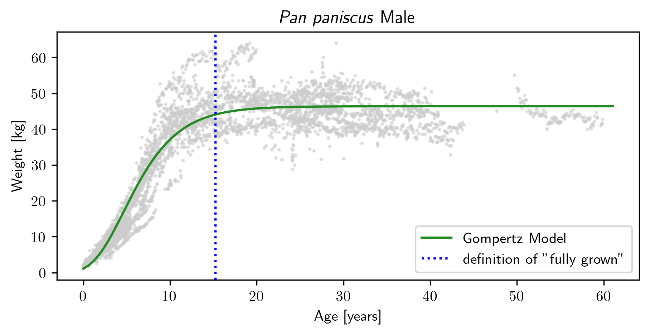 \| \| 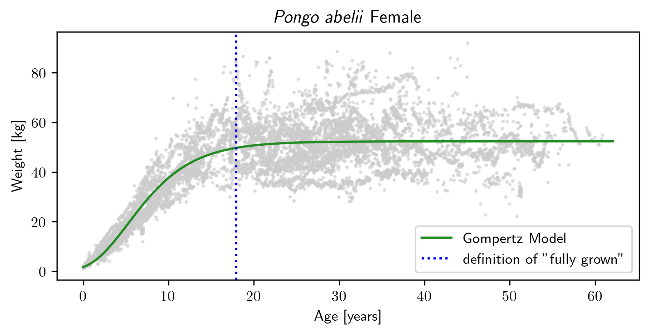 \| 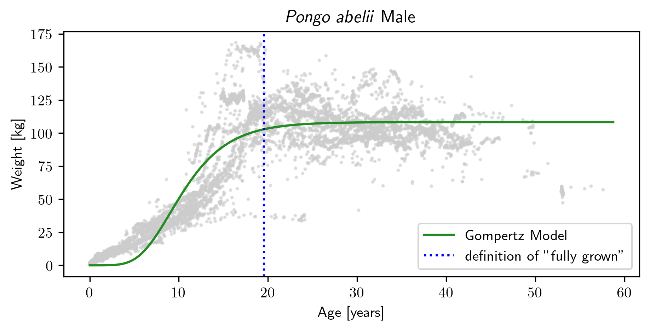 \| \| 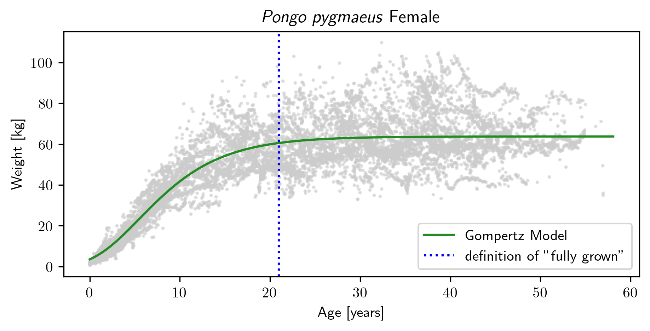 \| 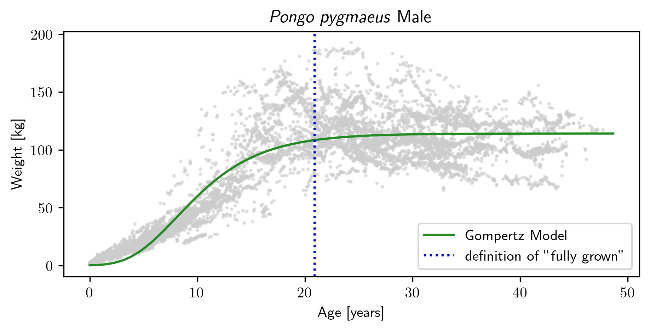 \| \| 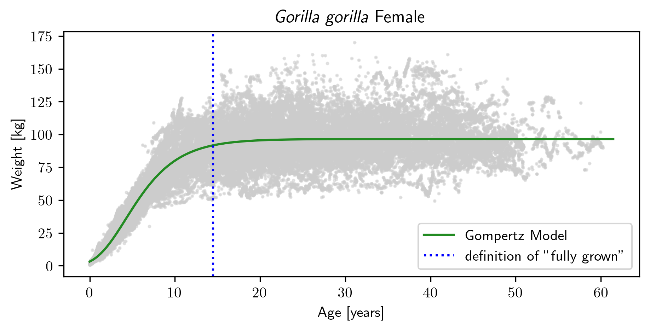 \| 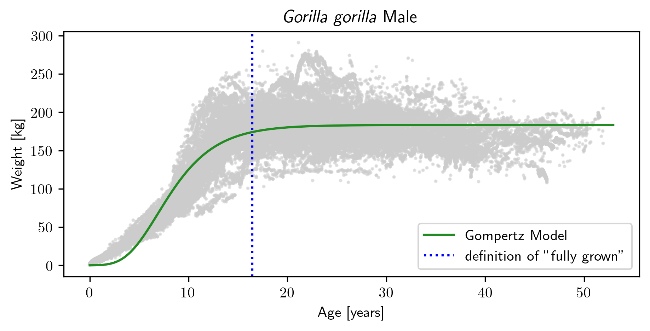 \| |
| --- | --- | --- | --- | --- | --- | --- | --- | --- | --- | --- |
|  |

**Figure S1** Gompertz growth models (green line) fitted to the age-specific body mass data of females and males of zoo-kept great apes. For a description of the model parameters, see Table 1.


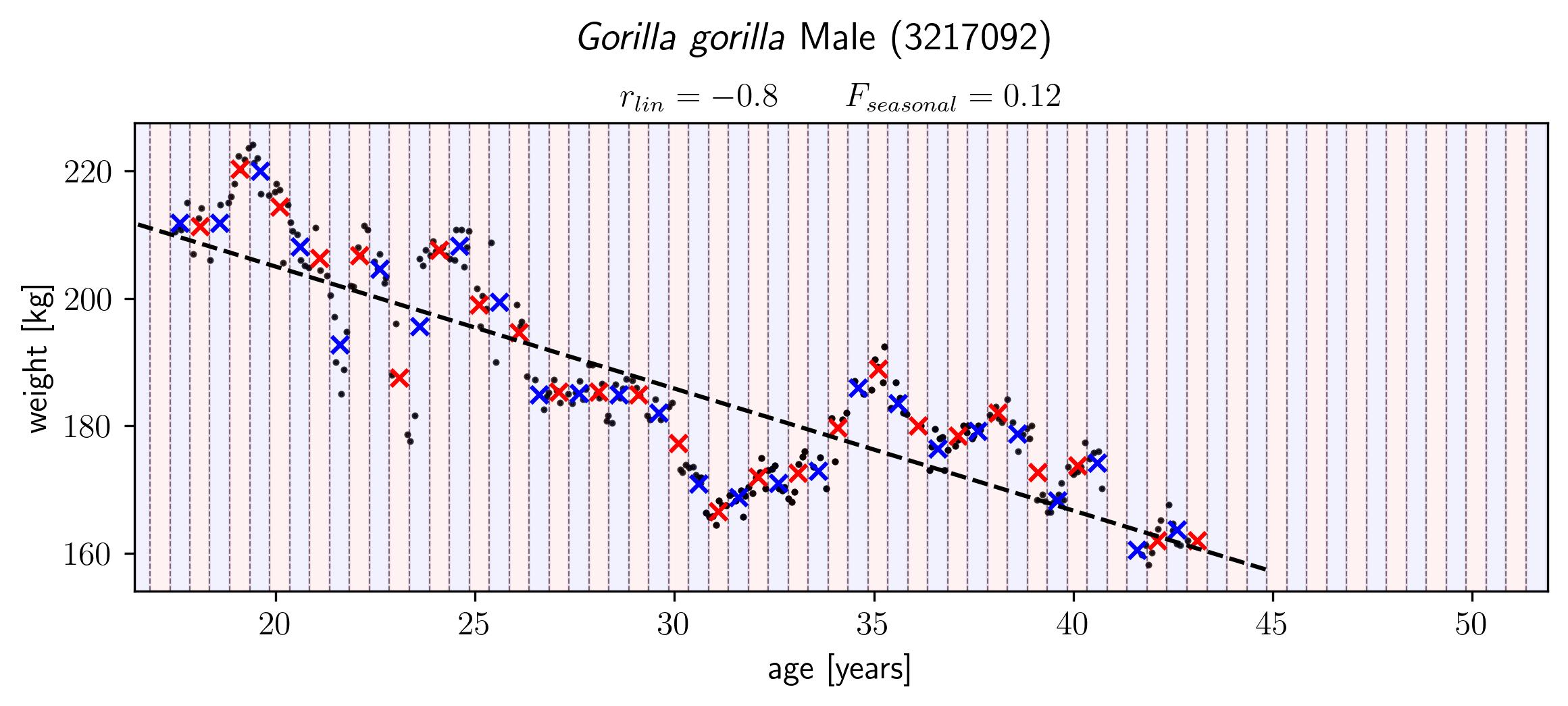


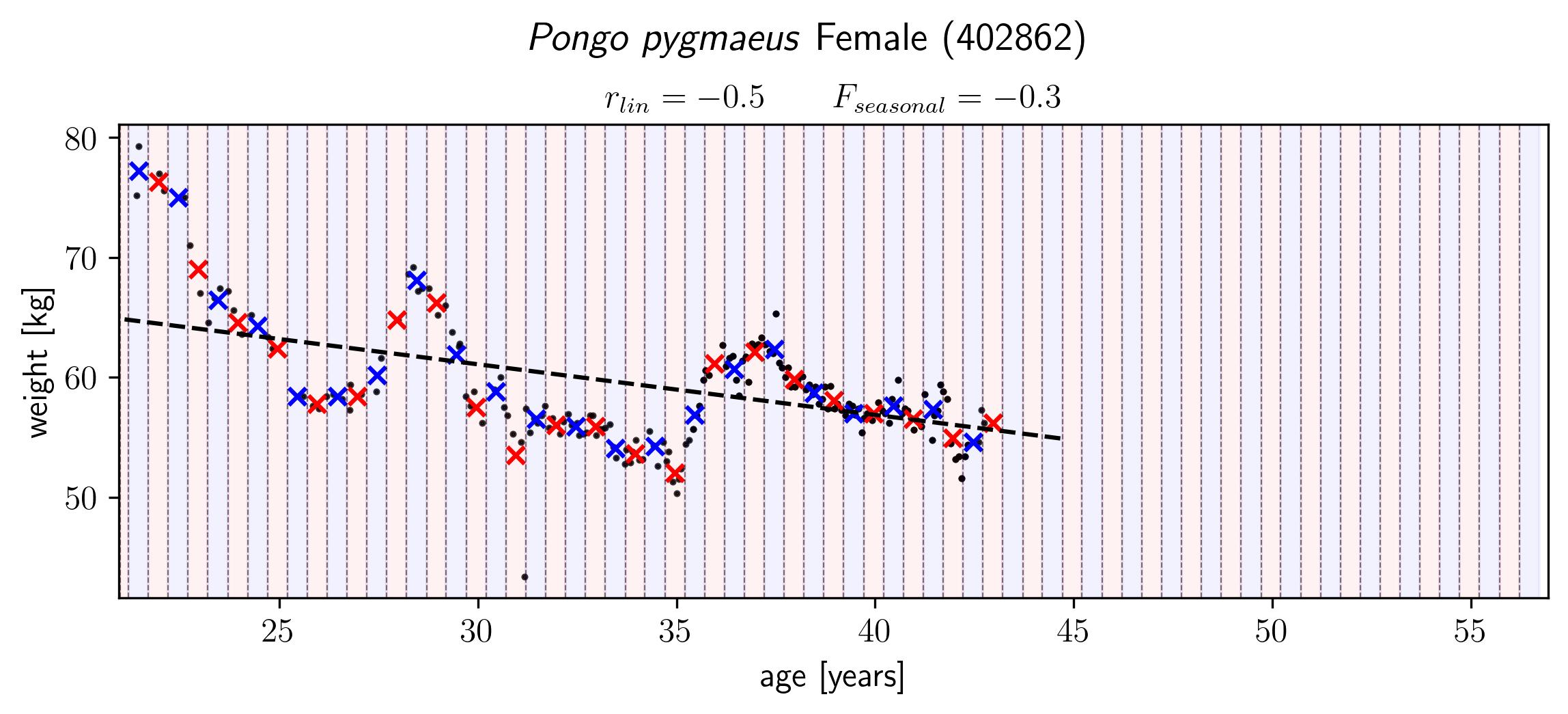


**Figure S2** Example for the visual display of body mass development in a male gorilla (top) and a female Bornean orangutan (bottom). Note a general trend of body mass decline with age (top), and an irregular change of body mass in cycles spanning several years (both).

|  |
| --- |

**Figure S3:** Highlighting of what is suspected to be Kanzi, a bonobo, renowned historically for a period of distinct obesity that was considered an outlier and removed from the dataset. The limited amount of data for this individual is due to its institution stopping collaboration with Species360. The reported weights are before its weight loss.


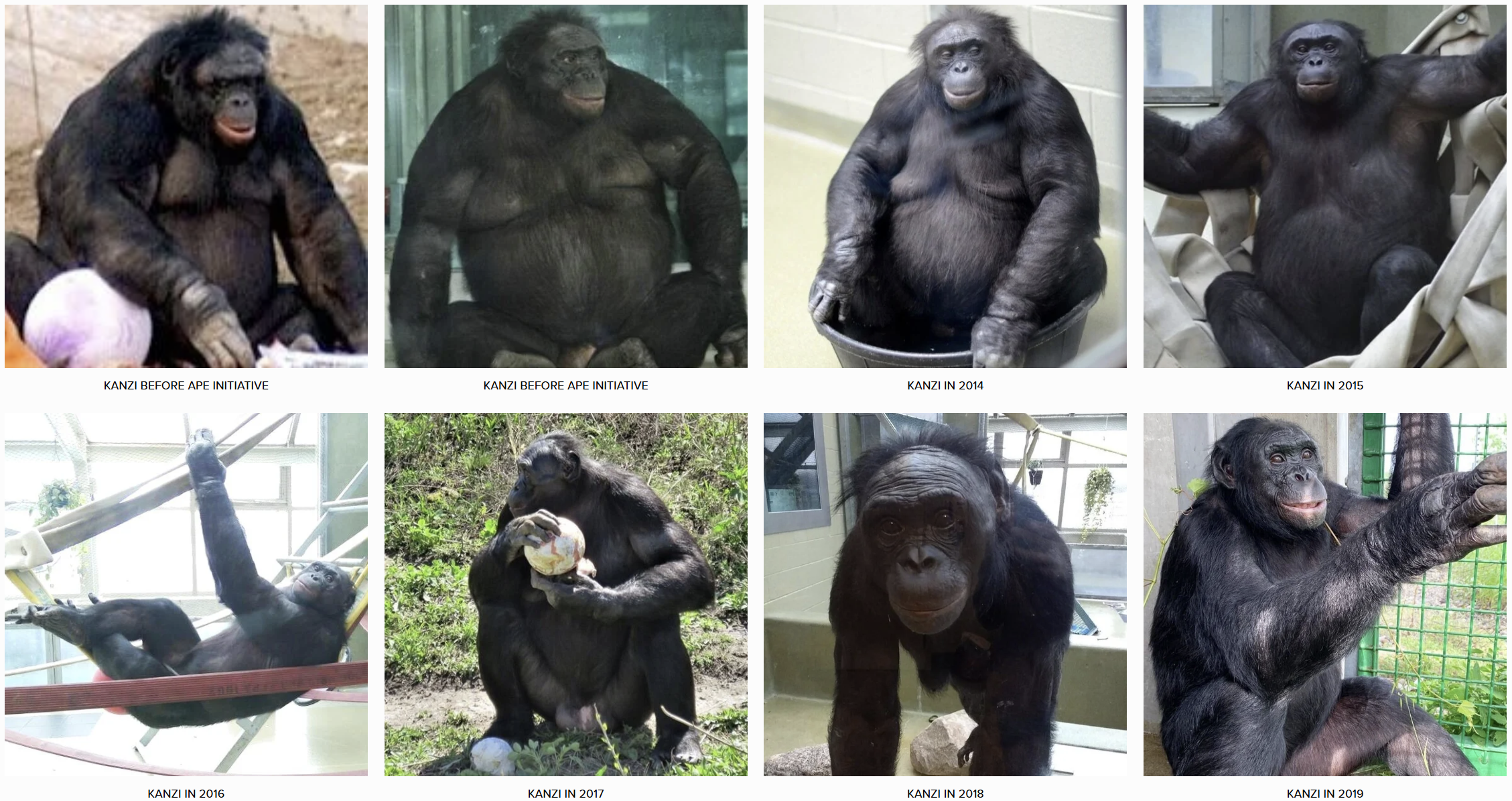


**Figure S4:** Body condition development of Kanzi over the course of more than five years. Source: <https://www.apeinitiative.org/kanzi>

| ***P. t. troglodytes*** |
| --- |
| **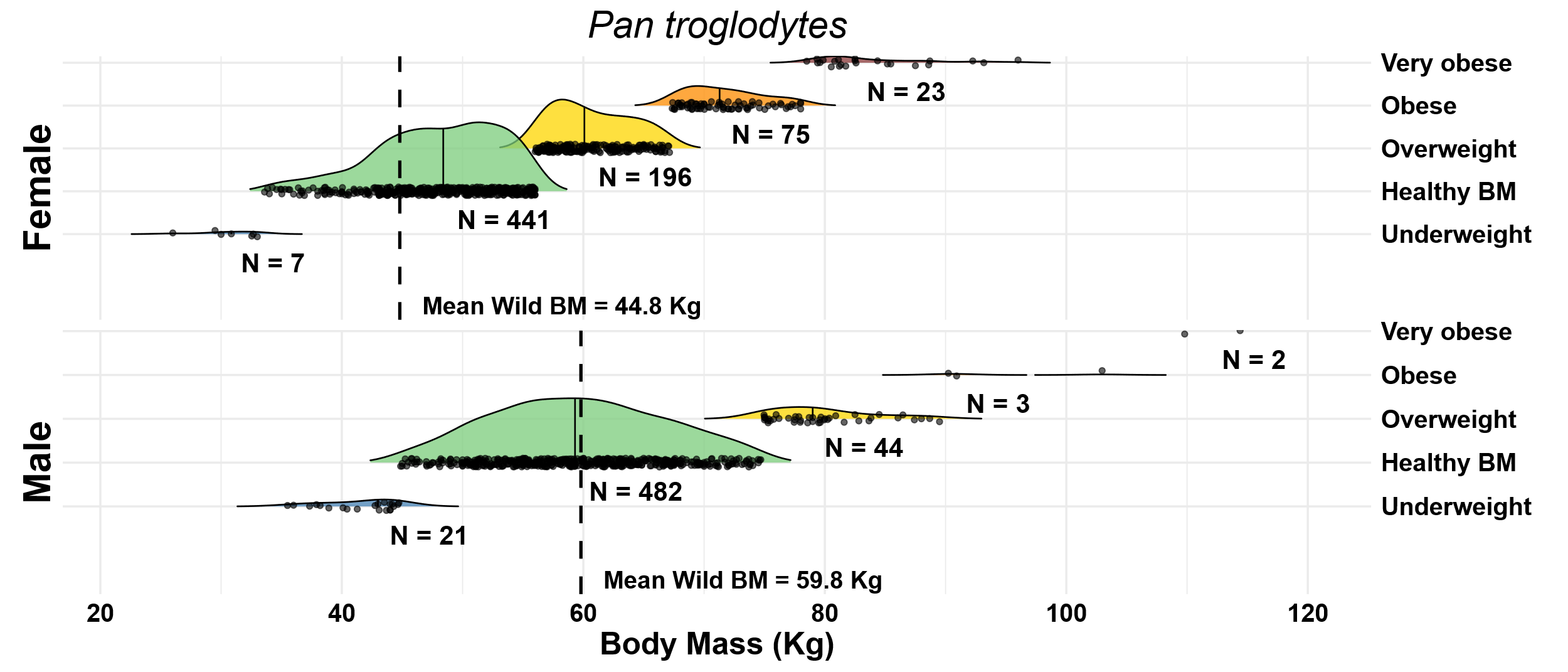** |
| ***P. t. verus*** |
| **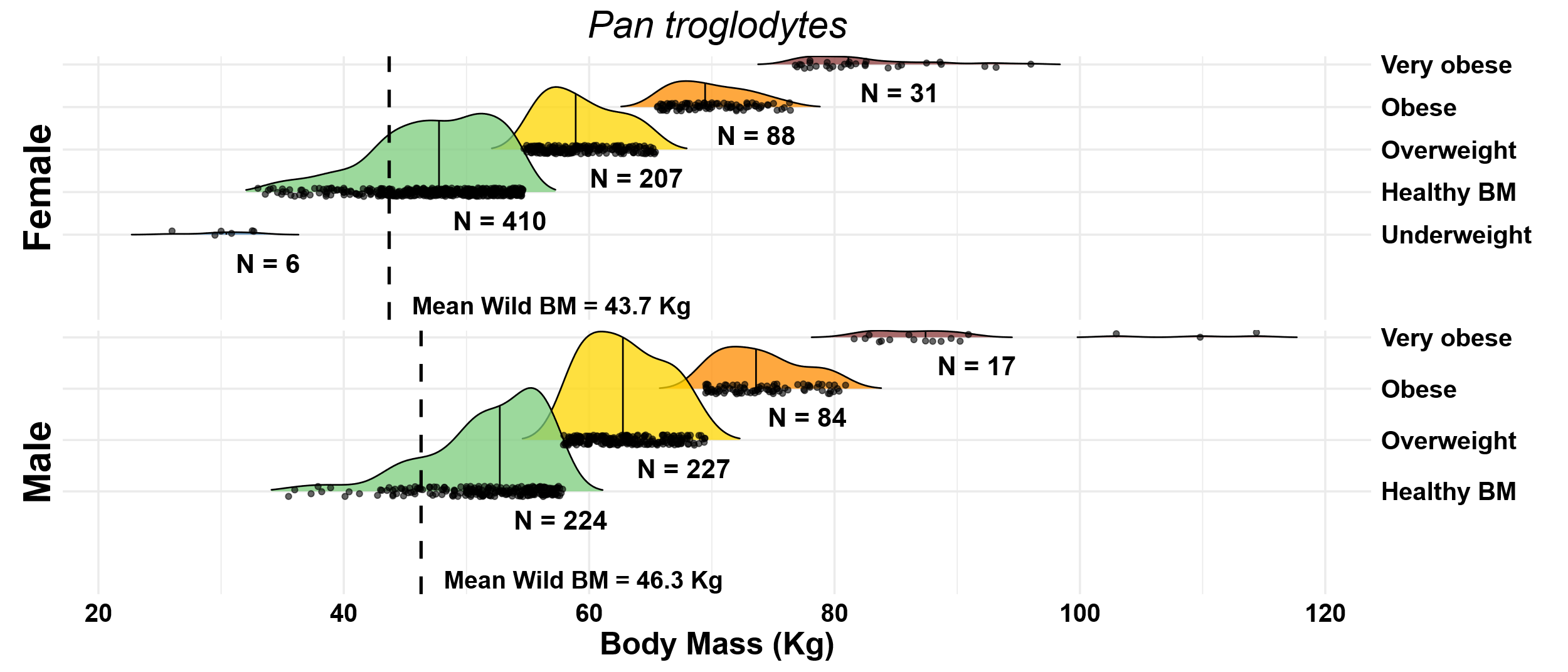** |
| ***P. t. schweinfurthii*** |
| **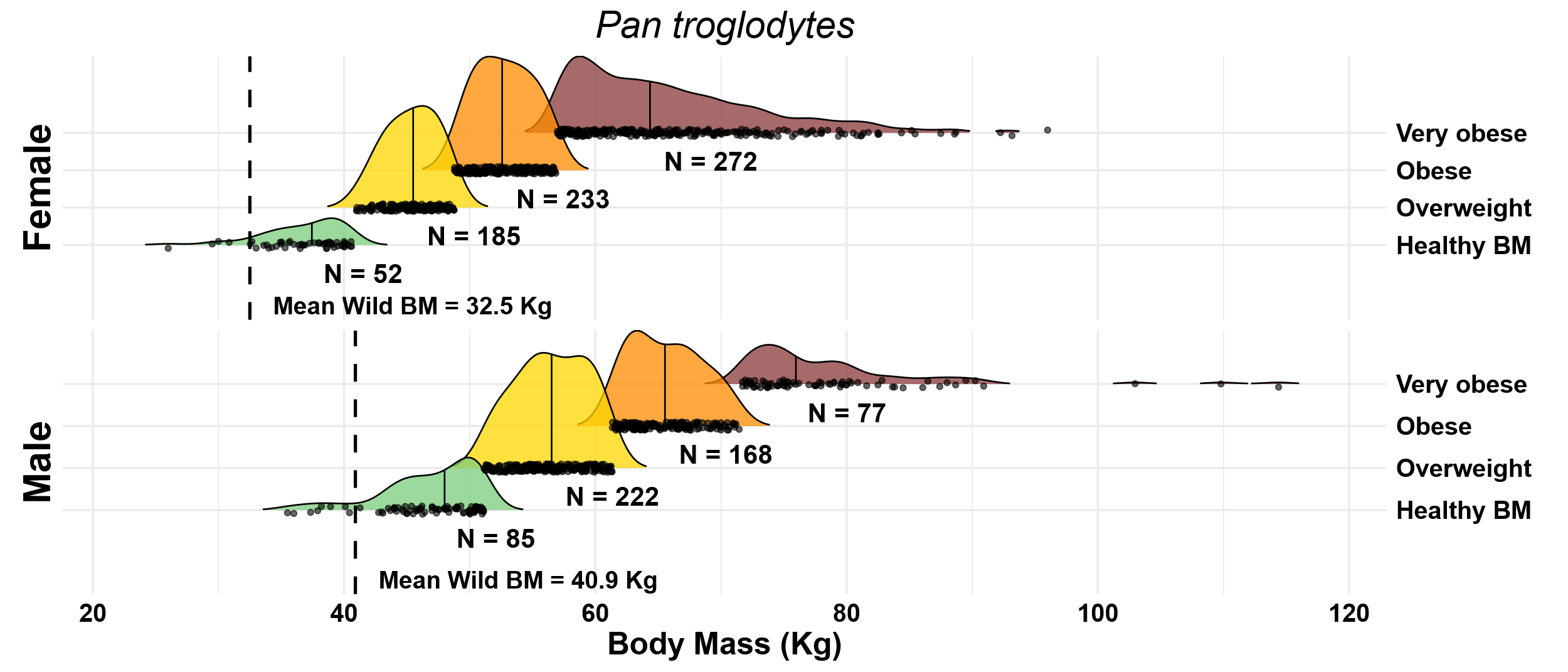** |

**Figure S5:** Distribution of densities of body mass scores of zoo chimpanzees if using each subspecies' average body mass as a benchmark.

References

Burgess, M. L., McFarlin, S. C., Mudakikwa, A., Cranfield, M. R., & Ruff, C. B. (2018). Body mass estimation in hominoids: Age and locomotor effects. *Journal of Human evolution*, *115*, 36-46. <https://doi.org/https://doi.org/10.1016/j.jhevol.2017.07.004>

Coolidge, H. J., & Shea, B. T. (1982). External body dimensions of *Pan paniscus* and *Pan troglodytes* chimpanzees. *Primates*, *23*(2), 245-251. <https://doi.org/10.1007/BF02381164>

Isler, K., Christopher Kirk, E., Miller, J. M. A., Albrecht, G. A., Gelvin, B. R., & Martin, R. D. (2008). Endocranial volumes of primate species: scaling analyses using a comprehensive and reliable data set. *Journal of Human evolution*, *55*(6), 967-978. <https://doi.org/https://doi.org/10.1016/j.jhevol.2008.08.004>

Jungers, W. L., & Susman, R. L. (1984). Body Size and Skeletal Allometry in African Apes. In R. L. Susman (Ed.), *The Pygmy Chimpanzee: Evolutionary Biology and Behavior* (pp. 131-177). Springer US. <https://doi.org/10.1007/978-1-4757-0082-4_7>

Lyon, M. W. (1911). Mammals collected by Dr. W. L. Abbott on Borneo and some of the small adjacent islands. *Proceedings of the United States National Museum*, *40*(1809), 53-146. <https://doi.org/10.5479/si.00963801.40-1809.53>

Markham, R., & Groves, C. P. (1990). Brief communication: Weights of wild orang utans. *American Journal of Physical Anthropology*, *81*(1), 1-3. <https://doi.org/https://doi.org/10.1002/ajpa.1330810102>

McLennan, M. R., & Asiimwe, C. (2016). Cars kill chimpanzees: case report of a wild chimpanzee killed on a road at Bulindi, Uganda. *Primates*, *57*(3), 377-388. <https://doi.org/10.1007/s10329-016-0528-0>

Morbeck, M. E., & Zihlman, A. L. (1989). Body size and proportions in chimpanzees, with special reference to *Pan troglodytes schweinfurthii* from Gombe National Park, Tanzania. *Primates*, *30*(3), 369-382. <https://doi.org/10.1007/BF02381260>

Pusey, A. E., Oehlert, G. W., Williams, J. M., & Goodall, J. (2005). Influence of ecological and social actors on body mass of wild chimpanzees. *International Journal of Primatology*, *26*(1), 3-31. <https://doi.org/10.1007/s10764-005-0721-2>

Rahm, U. (1967). Observations during chimpanzee captures in the Congo. *Progress in Primatology*. <https://cir.nii.ac.jp/crid/1570572700266212608>

Rayadin, Y., & Spehar, S. N. (2015). Body mass of wild Bornean orangutans living in human‐dominated landscapes: Implications for understanding their ecology and conservation. *American Journal of Physical Anthropology*, *157*(2), 339-346.

Smith, R. J., & Jungers, W. L. (1997). Body mass in comparative primatology. *Journal of Human evolution*, *32*(6), 523-559.

Uehara, S., & Nishida, T. (1987). Body weights of wild chimpanzees (*Pan troglodytes schweinfurthii*) of the Mahale Mountains National Park, Tanzania. *American Journal of Physical Anthropology*, *72*(3), 315-321.

Wich, S. A., Utami-Atmoko, S. S., Setia, T. M., Rijksen, H. D., Schürmann, C., van Hooff, J. A. R. A. M., & van Schaik, C. P. (2004). Life history of wild Sumatran orangutans (*Pongo abelii*). *Journal of Human evolution*, *47*(6), 385-398. <https://doi.org/https://doi.org/10.1016/j.jhevol.2004.08.006>

Wrangham, R. W., & Smuts, B. B. (1980). Sex differences in the behavioural ecology of chimpanzees in the Gombe National Park, Tanzania. *Journal of reproduction and fertility. Supplement*, *Suppl 28*, 13-31. <http://europepmc.org/abstract/MED/6934308>
